# Traditional Foods, Oral Microbiome, and Systemic Health: Molecular Pathways Linking Nutrition and Oral Disease Prevention

**DOI:** 10.3390/ijms27052412

**Published:** 2026-03-05

**Authors:** Juan Marcos Parise-Vasco, Jaime Angamarca-Iguago, Jaen Cagua-Ordoñez, Beatriz Cabrera, Dolores Jima Gavilanes, Raquel Horowitz, Claudia Reytor-González, Daniel Simancas-Racines

**Affiliations:** 1 Facultad de Ciencias de la Salud y Bienestar Humano, Universidad Tecnológica Indoamérica, Quito 170103, Ecuador; 2 Facultad de Medicina Veterinaria y Zootecnia, Universidad Agraria del Ecuador, Guayaquil 090104, Ecuador; gcabrera@uagraria.edu.ec; 3 Escuela de Medicina, Universidad Espíritu Santo, Samborondón 0901952, Ecuador; 4 Department of Medicine, Geriatrics Division, Montefiore Medical Center, Bronx, NY 10467, USA; rahorowitz@montefiore.org; 5 Facultad de Ciencias de la Salud y Bienestar Humano, Universidad Tecnológica Indoamérica, Ambato 180150, Ecuador

**Keywords:** oral microbiome, traditional diet, polyphenols, periodontal disease, systemic inflammation, dysbiosis, bioactive compounds, nutritional immunology

## Abstract

Periodontal disease affects 10–50% of the global population and is associated with various systemic conditions, including diabetes, cardiovascular disease and adverse pregnancy outcomes. Emerging evidence highlights diet as a critical, modifiable factor that influences the composition of the oral microbiome and periodontal health. This narrative review explores the molecular mechanisms through which traditional foods modulate the oral microbiome and contribute to oral and systemic health. A comprehensive literature search was conducted in PubMed/MEDLINE, the Cochrane Library, LILACS and Epistemonikos, prioritizing systematic reviews, meta-analyses and randomized controlled trials. The oral microbiome harbors over 700 bacterial species, and dysbiosis, characterized by pathogen enrichment, drives periodontal inflammation. Anti-inflammatory dietary patterns, including the Mediterranean diet, demonstrate protective effects. Omega-3 fatty acids, vitamins C and D, polyphenols and dietary fiber support periodontal health, whereas refined carbohydrates, saturated fats and pro-inflammatory nutrients can exacerbate disease. Probiotics show promise as an adjunctive therapy. However, the translation to clinical guidelines is impeded by methodological challenges, including the limited number of randomized controlled trials with oral endpoints, confounding by hygiene practices, and the lack of standardized multi-omics approaches. Nutritional counselling should be integrated into periodontal care as a modifiable risk factor. Future research priorities include precision nutrition approaches, the validation of salivary biomarkers, and interprofessional collaboration between dental and nutrition professionals.

## 1. Introduction

Oral health is an essential component of overall health and quality of life, yet oral diseases remain among the most prevalent non-communicable diseases globally [1]. Dental caries affects around 2.3 billion people worldwide, representing more than 25% of the global population. Severe periodontitis affects approximately 10% of the global population. These conditions, therefore, represent a significant public health burden [2,3]. The oral cavity harbors the second most diverse microbiota in the human body, comprising over 700 identified bacterial species, along with fungi, archaea, and viruses that colonize distinct ecological niches [1,4].

This intricate microbial consortium, collectively known as the oral microbiome, plays a vital role in maintaining oral homeostasis by interacting symbiotically with host tissues. However, it can transition to pathogenic configurations under specific environmental pressures [5]. The ecological plaque hypothesis, first proposed by Marsh and subsequently expanded upon [6,7], posits that oral diseases such as dental caries and periodontitis arise not from specific pathogens alone, but from ecological shifts within the microbial community driven by environmental perturbations. This conceptual framework has been refined by identifying keystone pathogens, such as *Porphyromonas gingivalis*, that manipulate host immune responses and cause community-wide dysbiosis even at low abundance [8].

The clinical importance of oral microbiosis extends far beyond the oral cavity. Robust epidemiological evidence demonstrates bidirectional associations between periodontal disease and major systemic conditions, including cardiovascular disease, type 2 diabetes mellitus, and metabolic syndrome [9]. The chronic inflammation characteristic of periodontitis contributes to the overall inflammatory burden in the body, while the translocation of oral pathogens into the bloodstream can directly affect distant organs [10]. Strong associations have been documented between periodontal disease and cardiovascular disease, with oral bacteria detected within atherosclerotic plaques [11,12,13]. The relationship between oral health and diabetes mellitus is bidirectional: poorly controlled diabetes increases susceptibility to periodontitis, while periodontal inflammation exacerbates insulin resistance [14,15,16]. Furthermore, oral dysbiosis has been associated with adverse pregnancy outcomes, respiratory infections, and even colorectal cancer through the oral-gut microbiome axis [14,15].

Current oral disease management strategies are primarily based on the mechanical removal of bacterial biofilms and the use of synthetic antimicrobial agents, such as chlorhexidine [1]. However, these approaches have limitations, including the development of antimicrobial resistance and adverse effects such as tooth staining and altered taste perception. There is also the challenge of achieving long-term compliance [17,18]. Consequently, there is growing interest in exploring complementary and alternative approaches based on traditional diet practices. For millennia, diverse cultures have utilized specific foods and plant-based remedies to maintain oral health and treat oral ailments [19]. Modern scientific research now validates many of these traditional practices, identifies the bioactive compounds responsible for their therapeutic effects, and elucidates their molecular mechanisms of action.

Traditional diets characterized by the high consumption of minimally processed whole foods, such as fruits, vegetables, legumes, herbs, and spices, are rich in polyphenols, flavonoids, terpenoids and other bioactive molecules with antimicrobial, anti-inflammatory, and antioxidant properties [17,19]. These compounds can modulate the composition of the oral microbiome, inhibit key pathogenic processes such as biofilm formation and expression of virulence factors, and dampen the host’s destructive inflammatory response [20,21]. Dietary interventions are an attractive approach to the prevention and management of oral diseases because they are generally safe, culturally acceptable, and cost-effective and can provide benefits to health beyond the mouth [22].

Contemporary research has begun to elucidate the effects of dietary components on oral microbial communities and host responses; however, significant knowledge gaps persist regarding the specific molecular pathways linking dietary bioactive compounds to modulation of the oral microbiome and subsequent systemic health outcomes [23]. For example, refined carbohydrates typical of Western dietary patterns have been shown to increase gingival inflammation, while traditional diets rich in complex carbohydrates can reduce the risk of gingivitis and periodontitis [24]. Clinical evidence suggests that the adoption of anti-inflammatory dietary patterns can reduce gingival bleeding indices by up to 40%, indicating meaningful clinical benefits of dietary modification [25]. In particular, dietary fiber has been shown to significantly affect the beta diversity of the oral microbiome and reduce potentially pathogenic taxa while promoting beneficial species [23]. However, beta diversity is an abstract measure of the relative abundances of species present and lacks a direct association with molecular mechanisms. Moreover, even the characterization of beneficial or pathogenic species requires complete and accurate genomic references and functional annotations. Recent reviews have revealed that 30–77% of oral microbial species lack reference genomes, limiting functional characterization [10] and indicating that the transition from descriptive community profiling to a mechanistic understanding of the interactions between diet, the microbiome and the host remains incomplete [26]. This review, therefore, aims to explore the available evidence on molecular mechanisms through which traditional foods and their bioactive compounds modulate the oral microbiome and contribute to oral and systemic health.

## 2. Methods

This narrative review explores the available literature on the relationship between traditional foods, oral microbiome modulation, and periodontal health. A comprehensive literature search was conducted in PubMed/MEDLINE, the Cochrane Library, LILACS, and Epistemonikos, using a combination of related search terms, including “periodontal disease”, “periodontitis”, “gingivitis”, “oral microbiome”, “oral microbiota”, “subgingival microbiome”, “diet”, “nutrition”, “dietary patterns”, “Mediterranean diet”, “omega-3 fatty acids”, “vitamin C”, “vitamin D”, “antioxidants”, “polyphenols”, “dietary fiber”, “probiotics”, “prebiotics”, “inflammation”, “dysbiosis”, and “host-microbiome interactions”. The study considered original research articles, reviews, meta-analyses, and clinical practice guidelines that addressed the influence of dietary components on oral microbiome composition or periodontal outcomes. Reference lists of key articles were manually screened to identify additional relevant studies. As this is a narrative review, no formal study selection protocol, risk-of-bias assessment, or quantitative synthesis was performed. Instead, the identified literature was critically appraised and thematically organized to provide an integrated overview of the molecular pathways linking nutrition, the oral microbiome, and oral disease prevention. For the purposes of this review, the term ‘traditional foods’ refers to the full range of whole foods and beverages that are consumed as part of established cultural dietary practices, such as green tea, turmeric, olive oil and fermented soy products. It also refers to the specific bioactive compounds that are responsible for their biological effects, such as EGCG, curcumin and hydroxytyrosol. Additionally, it refers to traditional preparations, such as essential oils and herbal extracts, including oregano oil and propolis. While Section 4 examines these foods and preparations in their cultural and dietary contexts, Section 5 extends the analysis to the molecular level to discuss the mechanisms of individual bioactive compounds. Selected compounds from traditional herbal medicine (e.g., baicalin and berberine) and pharmacological comparators are included to illustrate shared or contrasting molecular pathways. However, these are not traditional foods themselves.

## 3. The Oral Microbiome as a Gatekeeper

### 3.1. Composition and Ecological Organization

The human oral cavity represents one of the most complex and accessible microbial ecosystems in the body, hosting a remarkably diverse community of microorganisms. This ecosystem comprises more than 700 identified bacterial species, in addition to fungi, archaea, viruses, and bacteriophages [1,2]. The oral microbiome is not uniformly distributed throughout the human oral cavity; rather, it is organized into distinct ecological niches, each with unique environmental characteristics that select specific microbial communities [4,21]. These niches include the hard, non-shedding surfaces of teeth (supragingival and subgingival plaque), the soft mucosal tissues (tongue, buccal mucosa, hard and soft palate), and the specialized environment of the gingival sulcus, which represents an interface between the tooth surface and the host tissues [21].

The bacterial component of the oral microbiome is dominated by several major phyla, including *Firmicutes*, *Bacteroidetes*, *Proteobacteria*, *Actinobacteria*, *Spirochaetes* and *Fusobacteria* [2]. Within these phyla, certain genera consistently emerge as core members of the oral community between individuals. Key genera include *Streptococcus*, *Veillonella*, *Actinomyces*, *Fusobacterium*, *Prevotella*, *Neisseria*, *Haemophilus*, and *Porphyromonas* [1]. The early colonizers of clean tooth surfaces are predominantly aerobic and facultative anaerobic Gram-positive cocci, particularly species of Streptococcus such as *S. sanguinis*, *S. mitis*, and *S. salivarius* [1,27]. These pioneer species create the foundation for the subsequent succession of microbials. As the biofilm matures, the local environment becomes increasingly anaerobic, allowing the establishment of more fastidious anaerobic species [10].

*Actinomyces* species, particularly *A. naeslundii*, are Gram-positive rods that play an important role in early plaque formation and demonstrate remarkable metabolic flexibility; *Fusobacterium nucleatum* is a Gram-negative anaerobic species of particular interest due to its role as a “bridge organism” in biofilm development [1]. *F. nucleatum* has numerous adhesins that allow it to coaggregate with early colonizers and late colonizers, effectively linking different stages of biofilm maturation [1,2]. The *Veillonella genus*, comprising small Gram-negative anaerobic cocci, metabolizes lactate produced by streptococci, thus helping to moderate pH in dental biofilms [1].

Beyond bacteria, the oral mycobiome is dominated by the genus *Candida*, with *Candida albicans* being the most prevalent fungal species [1]. Fungi can interact synergistically with bacteria, influencing biofilm architecture and resilience [21]. Archaea, particularly methanogens from the phylum *Euryarchaeota*, are present in the oral cavity and have been found in greater abundance in individuals with periodontitis, although their precise ecological role remains under investigation [21,28]. The oral virome consists of both eukaryotic viruses and a wide diversity of bacteriophages that can modulate bacterial populations and facilitate horizontal gene transfer, thereby influencing the genetic plasticity and adaptability of the oral microbiome [21].

### 3.2. Functional Roles in Maintaining Oral Homeostasis

A balanced and diverse oral microbiome performs several critical functions that are essential to maintain oral health. One of its primary protective functions is colonization resistance, in which the resident commensal microbiota occupies available ecological niches and competes with exogenous pathogens for adhesion sites, nutrients, and space [1]. This competitive exclusion creates a biological barrier that prevents the establishment of foreign pathogens. Commensal bacteria also produce antimicrobial substances, including bacteriocins and hydrogen peroxide, which can inhibit the growth of pathogenic species. For example, *Streptococcus salivarius* produces salivaricin, a bacteriocin that targets other streptococcal species, thus regulating the structure of the microbial community [29].

The oral microbiome is intimately involved in immune modulation and host education. The constant interaction between commensal microorganisms and the host immune system is essential for the development and maturation of local immune responses [2]. This continuous cross-talk helps establish a state of immune tolerance to commensal organisms while maintaining the capacity to mount robust responses against true pathogens. Commensals can induce the production of secretory IgA and stimulate regulatory T cells, which suppress excessive inflammatory responses [1,2]. This delicate balance is crucial; an overly aggressive immune response to commensals can lead to inflammatory disease, while an insufficient response can allow pathogens to thrive.

The oral microbiota also performs important metabolic functions with systemic implications. A particularly notable example is the metabolism of dietary nitrate [1]. Nitrate-reducing bacteria, particularly facultative anaerobes in the tongue dorsum, convert dietary nitrate (abundant in leafy green vegetables) into nitrite. This nitrite can be further reduced to nitric oxide (NO) in acidic environments, such as the stomach or by host tissues. NO is a potent vasodilator and plays a critical role in blood pressure regulation, cardiovascular function, and immune signaling [30]. This enterosalivary nitrate-nitrite-NO pathway represents a direct mechanistic link between the oral microbiome, diet, and cardiovascular health. Disruption of this pathway, such as by using antiseptic mouthwashes that indiscriminately kill oral bacteria, has been associated with increased blood pressure [1,2].

### 3.3. Oral Dysbiosis and Disease Pathogenesis

Oral dysbiosis represents a pathological shift in the composition, diversity and function of the oral microbiome, moving from a state of symbiotic balance to one that promotes disease [31]. This ecological transition is not random but driven by environmental pressures such as dietary changes (particularly high sugar consumption), poor oral hygiene, smoking, host immune dysfunction, and alterations in salivary composition [1]. Dysbiosis is central to the pathogenesis of the two most prevalent oral diseases: dental caries and periodontitis.

The development of dental caries is best explained by the hypothesis of ecological plaques, which posits that caries is not caused by a single pathogen but rather results from an ecological shift within the dental biofilm driven by frequent exposure to fermentable carbohydrates [32]. In terms of health, the biofilm maintains a neutral pH. However, frequent consumption of dietary sugars leads to rapid fermentation by acidogenic bacteria, which causes the pH of the biofilm to drop below the critical threshold of approximately 5.5, at which point enamel demineralization begins [1]. This acid environment selects aciduric (acid-tolerant) species, most notably *Streptococcus mutans*, which can produce and tolerate high concentrations of lactic acid [21,32].

*S. mutans* is considered the primary etiological agent of dental caries due to its three key virulence factors: acidogenicity (the ability to rapidly produce acid from sugars), aciduricity (the ability to survive and continue metabolizing in low pH), and the capacity to synthesize extracellular polysaccharides (EPS) [21,33]. The enzyme glucosyltransferase (Gtf), particularly GtfB and GtfC, catalyzes the synthesis of sticky water-insoluble glucans from sucrose [10,34]. These glucans mediate bacterial adhesion to the tooth surface and create a protective matrix that shields bacteria from host defenses and antimicrobial agents [33]. Over time, prolonged acid exposure within this protected microenvironment leads to progressive demineralization of enamel and dentin, culminating in cavitation.

Periodontitis is a chronic inflammatory disease characterized by progressive destruction of the tooth-supporting structures: gingival tissue, periodontal ligament, and alveolar bone [28,35]. The disease is initiated by a shift from a health-associated microbiome dominated by Gram-positive facultative anaerobes to a dysbiotic community enriched in Gram-negative anaerobic bacteria [28]. A specific consortium known as the “red complex”—*Porphyromonas gingivalis*, *Tannerella forsythia*, and *Treponema denticola*—has been strongly associated with severe periodontitis [28,35].

*P. gingivalis* is considered a ‘keystone pathogen’, and even at relatively low levels, it can cause a shift towards dysbiosis in the entire community and disrupt the host immune response [22]. It produces a variety of virulence factors, including gingipains (cysteine proteases), lipopolysaccharide (LPS), and fimbriae, which allow it to invade epithelial cells, evade immune clearance, and promote a chronic inflammatory state [28,35]. The host inflammatory response to these pathogens paradoxically drives tissue destruction through the release of pro-inflammatory cytokines and matrix metalloproteinases, creating a self-reinforcing cycle of inflammation and tissue loss

### 3.4. The Oral-Systemic Health Connection

The oral cavity is not an isolated system; it serves as a portal to the rest of the body, and oral health status has profound implications for systemic health. The mechanisms underlying the oral-systemic health axis include (1) bacteremia—the translocation of oral pathogens and their virulence factors into the bloodstream through ulcerated periodontal tissue, (2) dissemination of local inflammation to become systemic, contributing to a chronic low-grade inflammatory state, and (3) shared risk factors such as smoking, poor diet, and stress that predispose to oral and systemic diseases [2,10].

The association between periodontitis and cardiovascular disease (CVD) is one of the most extensively studied oral–systemic connections. Individuals with periodontitis have a significantly elevated risk of atherosclerosis, myocardial infarction, and stroke [11], and DNA from oral pathogens has been detected within atherosclerotic plaques, suggesting direct involvement in atherogenesis [11]. The systemic inflammatory burden generated by periodontitis promotes endothelial dysfunction and a pro-thrombotic state [11,36].

The relationship between oral health and diabetes is bidirectional: poorly controlled diabetes exaggerates inflammatory responses to periodontal bacteria through hyperglycemia-driven neutrophil dysfunction and advanced glycation end product (AGE) formation [14,36], while chronic periodontal inflammation can worsen insulin resistance [36]. Successful periodontal treatment has been shown to improve HbA1c levels in diabetic patients [36,37], and recent Mendelian randomization studies support a causal link between specific oral bacterial taxa and the risk of type 2 diabetes [37,38].

Periodontal disease has been associated with adverse pregnancy outcomes, including preterm birth, low birth weight, and preeclampsia [39,40]. The proposed mechanism involves the hematogenous spread of oral pathogens, particularly F. nucleatum, to the placental-fetal unit, where they can induce inflammatory responses that can trigger premature labor [40,41]. Pregnant women experience physiological changes in hormones and immunity that can exacerbate gingival inflammation, highlighting the importance of maintaining oral health during pregnancy.

Daily swallowing of saliva results in continuous translocation of oral bacteria to the gastrointestinal tract, estimated at 1012 bacteria per day [40]. Under normal conditions, gastric acid provides a formidable barrier for most oral microbes. However, in individuals who use proton pump inhibitors or those with gastric pathology, oral bacteria can survive passage to the intestines and establish ectopic colonization [40,41]. Oral species such as *F. nucleatum* and *P. gingivalis* have been found to be enriched in the gut mucosa of patients with inflammatory bowel disease and colorectal cancer, where they can contribute to intestinal inflammation and tumor progression [40,42]. This oral-gut microbiome axis represents a critical pathway through which oral dysbiosis can have profound effects on systemic health.

### 3.5. Comparison with the Gut Microbiome

Although oral and gut microbiomes are anatomically connected through the gastrointestinal tract, they are ecologically and compositionally distinct ecosystems [40]. The oral cavity is characterized by constant exposure to the external environment, mechanical forces from mastication, and high salivary flow, while the intestinal tract features a steep oxygen gradient, varying pH levels, bile acids, and a mucosal immune system [40,43]. Compositionally, the oral microbiome exhibits higher alpha diversity at the species level, but lower biomass compared to the gut. The gut microbiome is overwhelmingly dominated by two phyla, *Bacteroidetes* and *Firmicutes*, which together comprise more than 90% of the community, with much lower relative abundances of *Proteobacteria* and *Actinobacteria* [40,44]. In contrast, the oral microbiome shows a more balanced representation of multiple phyla.

Despite these differences, there is dynamic bidirectional communication between the two ecosystems. Oral commensals can influence gut health through the production of metabolites and immune priming, while gut-derived metabolites such as short-chain fatty acids can affect oral tissues [40]. Dysbiosis in one site can influence the other, as evidenced by the increased presence of oral pathobionts in the intestinal tract of individuals with inflammatory bowel disease [27]. This interconnectedness underscores the importance of a holistic approach to health that considers the microbiome as an integrated system spanning multiple body sites [45].

## 4. Traditional Foods and Their Bioactive Compounds

Traditional diets from various cultures contain a wide range of foods that are rich in bioactive compounds with therapeutic potential for oral health. Consumed for centuries, these foods are now being scientifically validated for their antimicrobial, anti-inflammatory, and antioxidant properties. This section examines traditional foods from major geographic regions, focusing on their key bioactive constituents and the mechanisms by which they prevent oral disease.

### 4.1. Traditional Asian Foods

Green Tea and Epigallocatechin-3-Gallate (EGCG): Green tea (*Camellia sinensis*) is a cornerstone of traditional Asian medicine and has been consumed for millennia for its health-promoting properties [46,47]. Its primary bioactive constituents are polyphenolic catechins, with EGCG being the most abundant and biologically active, comprising 40–48% of total catechins [46]. EGCG demonstrates broad-spectrum antimicrobial activity against oral pathogens through multiple mechanisms [46,48]. It disrupts bacterial cell membranes by embedding into the lipid bilayer, causing transverse expansion and eventual rupture [48]. EGCG also targets the virulence factors of *S. mutans*, inhibiting critical enzymes including glucosyltransferases (Gtfs), F1F0-ATPase, lactate dehydrogenase, and the phosphoenolpyruvate-dependent phosphotransferase system [33]. By inhibiting Gtfs, EGCG prevents the synthesis of sticky glucan polymers essential for bacterial adhesion and biofilm integrity [33]. Against periodontal pathogens such as *P. gingivalis*, *Prevotella intermedia*, and *F. nucleatum*, EGCG exhibits even more potent bactericidal effects [45,49]. Studies have shown that EGCG exhibits antimicrobial activity against periodontal bacteria at concentrations ranging from 0.019 to 1.25 mg/mL (MIC), with clinical trials demonstrating that 5 mg/mL EGCG as an adjunct to scaling and root planing significantly reduces probing depth, improves clinical attachment level, and decreases periodontal pathogen load over 3–6 months, suggesting strong potential for periodontitis management [50,51,52].

Fermented Soy (Natto), a traditional Japanese food made from fermented soybeans with *Bacillus subtilis natto*, possesses remarkable anti-biofilm properties [53]. Research has demonstrated that natto effectively inhibits the formation of sucrose-dependent biofilms by cariogenic streptococci, particularly *S. mutans* and *S. sobrinus* [54]. The mechanism is primarily attributed to proteolytic enzymes in natto, with characteristics similar to nattokinase [55]. These proteases specifically target the production of water-insoluble glucan by *S. mutans* without necessarily reducing bacterial viability, suggesting an anti-virulence rather than bactericidal mode of action [53,54]. Additionally, *B. subtilis* natto itself exhibits probiotic properties, showing strong adherence to extracellular matrix proteins and producing natural antimicrobial compounds active against pathogenic bacteria and fungi [54].

Turmeric (Curcumin), a staple spice in Asian cuisine and traditional medicine, contains curcumin as its primary bioactive polyphenol [21]. Curcumin exerts potent anti-inflammatory effects through multiple pathways. It binds to Toll-like receptors (TLRs) and modulates downstream signaling cascades, including NF-κB, MAPK, and AP-1 [21,56]. Curcumin downregulates NF-κB through interaction with PPARγ and regulates the JAK/STAT pathway [20]. It directly inhibits the activation of the NLRP3 inflammasome and decreases the production of pro-inflammatory mediators, including IL-1β, IL-6 and TNF-α [20,56]. In the oral context, studies have shown that curcumin can significantly decrease both plaque index and gingival index with efficacy comparable to chlorhexidine, but with fewer side effects [17]. Curcumin also demonstrates antibacterial properties against *S. mutans* by interfering with metabolic pathways and downregulating the *atpH* gene, which encodes a subunit of the F-ATPase proton pump essential for acid tolerance [21,57].

### 4.2. Traditional Mediterranean Foods

Extra virgin olive oil (EVOO) is a fundamental component of the Mediterranean diet, with its health benefits largely attributed to phenolic compounds, particularly hydroxytyrosol (HT) [58,59]. HT is one of the most potent natural antioxidants, with its hydroxylated phenolic ring structure that allows effective scavenging of various free radicals [59]. The European Food Safety Authority has officially recognized a health claim for olive oil polyphenols, acknowledging their role in protecting LDL from oxidative damage [60]. This potent antioxidant capacity is highly relevant for oral health, as oxidative stress is a major contributor to the pathogenesis of periodontal disease, causing damage to gingival tissues and alveolar bone [61]. Although research on HT’s direct impact on oral health parameters is still developing, its well-documented antioxidant and anti-inflammatory properties provide a strong theoretical basis for benefits in mitigating oxidative stress and inflammation within the oral cavity [59,62,63].

The aromatic herbs of the Mediterranean region—oregano (*Origanum vulgare*), thyme (*Thymus vulgaris*) and rosemary (*Rosmarinus officinalis*)—are rich sources of essential oils with potent antimicrobial properties [64]. Essential oils of oregano and thyme demonstrate powerful, broad-spectrum antimicrobial activity against a wide range of pathogens, including oral bacteria [64]. Their efficacy is mainly attributed to high concentrations of phenolic monoterpenes, specifically carvacrol and thymol [64]. These compounds exert antimicrobial effects by disrupting the structural and functional integrity of the bacterial cell membrane, increasing permeability, and causing leakage of intracellular components and eventual cell death [64]. Comparative studies consistently show that oregano and thyme essential oils are more effective than many other plant-derived oils, demonstrating potency against Gram-positive bacteria such as *Staphylococcus aureus* and Gram-negative bacteria such as *Escherichia coli*, as well as multidrug-resistant clinical isolates [64,65]. Their activity against oral pathogens makes them promising candidates for natural oral care products [64].

### 4.3. Traditional South American Foods

Cocoa (*Theobroma cacao*) is valued for its high content of flavonoids and polyphenols with significant oral health properties [66]. Research has demonstrated that cocoa extract has antimicrobial activity against key oral pathogens, including *S. mutans* and *F. nucleatum* [17]. Studies show that cocoa extract can inhibit the growth, adherence, and biofilm formation of these bacteria in a dose-dependent manner [17,66]. It also reduces acid production by cariogenic species and inhibits the activity of glycosyltransferase enzymes, thus disrupting the formation of the plaque biofilm matrix [17]. Clinical research involving mouthwashes containing cocoa extract has reported reductions in salivary *S. mutans* counts and decreases in dental biofilm accumulation, supporting its potential as an anti-caries agent [17,66]. Additionally, cocoa has anti-inflammatory properties that can help manage gingival inflammation [17].

Propolis, a resinous substance collected by bees from various plant sources, is a complex natural product containing hundreds of bioactive compounds, including high concentrations of flavonoids and phenolic acids [19]. It has a long history of use in traditional medicine for its potent anti-inflammatory, antioxidant, and antimicrobial properties [19,67]. Propolis demonstrates broad-spectrum antimicrobial activity against a wide range of oral pathogens, including *S. mutans*, *P. gingivalis*, and *F. nucleatum* [68]. Its mechanism involves the disruption of microbial membranes and the inhibition of bacterial growth [56,68]. The specific chemical composition and antimicrobial potency of propolis can vary significantly depending on its geographical origin and botanical sources [69]. Studies have shown that propolis can effectively reduce plaque and gingival inflammation, inhibit bacterial fermentation and acid production, and disrupt biofilm formation [68]. It has been successfully incorporated into oral care products such as toothpastes and mouthwashes [19]. Although some studies suggest that propolis may not be as potent as conventional antibiotics against certain bacteria, its low toxicity and broad range of action make it an excellent candidate for use as an adjuvant therapy or preventive agent in oral care [67].

### 4.4. Traditional North American Foods

The North American cranberry (*Vaccinium macrocarpon*) is rich in a unique class of polyphenols known as A-type proanthocyanidins (PACs) [70,71]; unlike B-type PAC found in most other fruits, the A-type linkage in cranberry PACs is believed crucial for their characteristic biological activity, particularly anti-adhesion properties [71]. Cranberry PACs have demonstrated a significant ability to interfere with the pathogenic processes of key oral bacteria, most notably *S. mutans* and *P. gingivalis* [72,73]. The primary mechanism is inhibition of bacterial adhesion and biofilm formation [74]. They effectively prevent *S. mutans* from adhering to tooth surfaces by inhibiting glucosyltransferases (Gtfs), the enzymes responsible for synthesizing the sticky glucan matrix that holds biofilms together [73,75]. By reducing the synthesis of insoluble glucans, PACs disrupt biofilm structural integrity and prevent bacterial aggregation [71].

Against periodontal pathogens, cranberry PACs neutralize various virulence factors of *P. gingivalis*, inhibiting its growth, reducing biofilm formation, and preventing its adhesion to and invasion of human oral epithelial cells in a dose-dependent manner [73,74]. Furthermore, cranberry PACs possess anti-inflammatory properties, inhibiting the secretion of pro-inflammatory signaling molecules such as IL-8 by oral epithelial cells stimulated by *P. gingivalis* [73]. This effect is associated with reduced activation of the NF-κB signaling pathway, a central regulator of inflammation [71,74]. Collective evidence strongly suggests that cranberry PACs are valuable bioactive molecules that can target both the microbial and inflammatory aspects of oral diseases [72], making them highly promising for the development of new preventive and therapeutic strategies for dental caries and periodontitis [70,71].

### 4.5. Middle Eastern Spices

The Middle Eastern cuisine is characterized by liberal use of aromatic spices with health-promoting and preservative properties [76]. Cardamom (*Elettaria cardamomum*) has been traditionally used as a breath freshener, and modern research supports its role in oral health [70,77]. Extracts from cardamom demonstrate antibacterial activity against major periodontal pathogens, including *Aggregatibacter actinomycetemcomitans* and *P. gingivalis* [78]. It also inhibits biofilm formation and significantly reduces secretion of inflammatory markers like IL-1β and TNF-α from immune cells, likely by inhibiting the NF-κB signaling pathway [70,78]. This dual antimicrobial and anti-inflammatory action makes it beneficial for managing periodontal infections [78,79].

**Figure 1:**
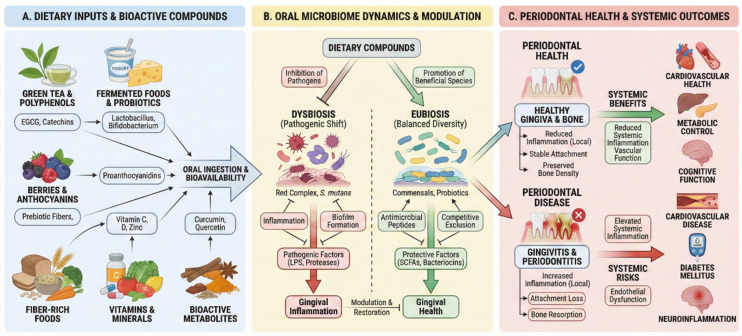
**Influence of Diet on Oral Microbiome and Systemic Health Outcomes.** This schematic illustrates the pathways through which dietary bioactive compounds influence periodontal and systemic health. (**A**) Main nutritional factors, including polyphenols, probiotics, prebiotic fibers, and micronutrients, and their effect on oral bioavailability. (**B**) Effects of these compounds on oral microbiome dynamics: promotion of eubiosis through competitive exclusion and production of protective factors (short-chain fatty acids, bacteriocins), or contribution to dysbiosis characterized by pathogenic biofilm formation and release of inflammatory mediators (lipopolysaccharides, proteases). (**C**) Bidirectional relationship between periodontal status and systemic health; periodontal disease amplifies systemic inflammation, endothelial dysfunction, and the risk of cardiovascular disease, diabetes mellitus, and neuroinflammation. Green arrows denote beneficial pathways; red arrows indicate pathological cascades [19,20,21,56,62,72,73,78,80,84]. Abbreviations: EGCG, epigallocatechin gallate; LPS, lipopolysaccharide; SCFAs, short-chain fatty acids; *S. mutans*, *Streptococcus mutans*. Created in BioRender. Reytor, C. (2026) https://BioRender.com/rfczwbs (accessed on 1 March 2026).

Cloves are perhaps one of the most well-known traditional remedies for dental ailments, particularly toothaches [56]. Their potent effects are primarily due to eugenol, which possesses powerful natural antiseptic, analgesic, and antibacterial properties [80]. Clove oil has been shown to be effective against the oral microbiota responsible for dental caries and helps reduce inflammation associated with gum disease [80,81]. Cinnamon also offers significant advantages for oral health, with cinnamon oil demonstrating broad-spectrum antibacterial activity against numerous bacterial species involved in dental caries, including a strong inhibitory effect on *S. mutans* [80,82]. Recent systematic reviews have concluded that herbal oral care interventions, including those containing spices such as clove, cinnamon, and other plant extracts, demonstrate comparable efficacy to conventional agents like chlorhexidine in improving periodontal status when used as adjuncts to scaling and root planing or supragingival debridement. Some individual herbal products showed superior results to placebo or non-surgical treatment alone, with few studies reporting better outcomes than chlorhexidine in specific parameters. However, the evidence indicates that herbal products generally perform similarly rather than superior to gold-standard antiseptics [83,84]. The complex interrelationships between dietary factors, oral microbiome dynamics, and periodontal health outcomes are illustrated in Figure 1.

## 5. Molecular Mechanisms of Action in the Oral Environment

The molecular mechanisms by which bioactive compounds from traditional foods modulate oral health have been elucidated primarily through in vitro studies and preclinical models. This section discusses the mechanisms of compounds derived directly from traditional foods (e.g., EGCG from green tea, curcumin from turmeric), as well as selected compounds from traditional herbal medicine and pharmacological agents that share or illuminate the same molecular pathways. While these investigations provide essential insights into the biochemical pathways involved, it is important to note that the level of evidence varies considerably across compounds: some, such as sub-antimicrobial dose doxycycline and curcumin, are supported by systematic reviews and clinical trials, whereas others, including carnosic acid and BML-111, have been investigated only in cell culture or animal models to date. Much of the mechanistic evidence discussed in this section derives from comprehensive reviews that synthesize findings from multiple study designs. Where evidence comes from a specific primary study, the study design is indicated. The clinical evidence for dietary interventions and functional foods is presented separately in Section 7.

### 5.1. Antibacterial Effects

*Streptococcus mutans* is the primary etiological agent of dental caries, with pathogenicity derived from acidogenicity, aciduricity, and EPS synthesis [21,33]. Bioactive compounds employ sophisticated anti-virulence strategies to neutralize these traits. EPS synthesis is mediated by glucosyltransferases (Gtfs), specifically GtfB, GtfC, and GtfD, which convert sucrose into sticky glucan polymers that form the structural scaffold of the biofilm matrix [10,34]. Natural compounds directly target this process. Cinnamaldehyde inhibits the expression of the vicR gene, a component of a two-component signal transduction system that regulates the transcription of *gtfB*, *gtfC* and *gtfD* [10]. Triterpenoid betulin also reduces the expression of these critical gtf genes [10,21,34]. EGCG from green tea directly affects the synthesis of biofilm matrix components, weakening biofilm structure [21,85].

Beyond inhibiting biofilm formation, bioactive compounds alter the acidogenic and aciduric capabilities of *S. mutans* [54,57,86,87]. Curcumin demonstrates potent antibacterial properties by interfering with metabolic pathways, including fatty acid, carbon, and pyruvate metabolism [21,57]. Crucially, curcumin downregulates the *atpH* gene encoding a subunit of the F-ATPase proton pump vital for the acid tolerance of *S. mutans*, as it expels excess protons from the cytoplasm to maintain a viable internal pH [21,54]. By impeding F-ATPase function, curcumin compromises the ability of the bacterium to survive in the acidic conditions it creates [21]. Farnesol further affects acid tolerance by enhancing proton flux across the bacterial membrane, thereby disrupting barrier function [21,34].

Disruption of quorum sensing (QS), the cell-to-cell communication system that bacteria use to coordinate group behaviors such as biofilm formation and virulence factor expression, represents another key anti-caries strategy [21,33]. In the context of *S. mutans*, compounds such as cinnamaldehyde and thymol downregulate QS-related genes, including *comDE* and *luxS*, effectively silencing signals that trigger pathogenic behavior [21,88,89]. To overcome challenges such as poor solubility and stability of these bioactive compounds, research is increasingly focusing on novel delivery systems, including nanoliposomes and chitosan hydrogels, to enhance oral bioavailability and sustained release [21,90].

In the context of periodontal disease, bioactive compounds target a distinct set of pathogens dominated by the red complex bacteria—*Porphyromonas gingivalis*, *Tannerella forsythia*, and *Treponema denticola*—as well as *Aggregatibacter actinomycetemcomitans* and *Fusobacterium nucleatum* [27,45]. Several distinct mechanisms of action have been identified. First, certain algal metabolites and lipopeptide-like compounds disrupt bacterial cell membranes by inserting into the lipid bilayer, compromising structural integrity and leading to pore formation, ion leakage, membrane depolarisation, and cell death [91,92]. Second, phlorotannins—polyphenols derived from marine algae—chelate metal ions essential for the catalytic activity of bacterial enzymes, thereby inhibiting key metabolic processes [45]. Third, quorum-sensing inhibition provides an anti-virulence strategy that reduces pathogenicity without directly killing bacteria, thus exerting less selective pressure for resistance development [21,93]; bromophenols, also derived from marine sources, have been noted for their potent QS inhibitory capacity [93,94]. Finally, bioactive compounds can directly disrupt the structure of mature biofilms by targeting the protective extracellular polymeric substance (EPS) matrix; for example, cationic dextrans induce phase transition in the EPS of *P. gingivalis* biofilms, destroying the gel-like structure and exposing encapsulated bacteria to antimicrobial agents and host defenses [95]. The efficacy of these approaches has been demonstrated in vitro, with naturopathic oral care products containing medicinal plant extracts and essential oils showing concentration-dependent antibacterial effects against *A. actinomycetemcomitans* and *F. nucleatum* [79,83,84].

### 5.2. Anti-Inflammatory Pathways

The NF-κB signaling pathway is a master regulator of the inflammatory response in virtually all cell types [34]. In periodontitis, bacterial components such as LPS from *P. gingivalis* act as potent activators via Toll-like receptor 4 (TLR4) [34,96]. In its inactive state, NF-κB is sequestered in the cytoplasm by its inhibitor IκB. Upon stimulation, the IκB kinase (IKK) complex phosphorylates IκB, marking it for ubiquitination and proteasomal degradation [34]. This unmasks a nuclear localization sequence on NF-κB, allowing its translocation to the nucleus [34]. Once in the nucleus, NF-κB binds to specific DNA sequences in promoter regions of target genes, driving transcription of pro-inflammatory mediators including IL-6, IL-1β, and TNF-α [97].

Bioactive compounds from natural sources modulate this pathway at multiple points along the signaling cascade. At the receptor level, baicalin, a flavonoid of *Scutellaria baicalensis*, inhibits the initial binding of LPS to its TLR4 receptor complex, preventing pathway activation [34]. At the cytoplasmic level, many phytochemicals prevent phosphorylation and subsequent degradation of IκB, trapping NF-κB in the cytoplasm and blocking its pro-inflammatory function. At the nuclear level, parthenolide targets the p65 subunit of NF-κB, suppressing its transcriptional activity even after nuclear entry [98]. Similarly, berberine promotes deacetylation of p65 at Lysine 310, highlighting the importance of epigenetic modifications in the control of inflammation. Compounds such as albiflorin and various polyphenols further demonstrate anti-inflammatory activity through NF-κB pathway inhibition, making this cascade a central therapeutic target for the management of oral inflammation [34,99]. Compounds such as albiflorin and various polyphenols further demonstrate anti-inflammatory activity through NF-κB pathway inhibition, making this cascade a central therapeutic target for the management of oral inflammation [100].

Gingival fibroblasts (GFs), the most abundant cell type in gingival connective tissue, are active participants in periodontal inflammation [101]. When stimulated by bacterial products, GFs produce TNF-α and IL-1β, which act in an autocrine and paracrine fashion to amplify inflammatory responses and regulate the expression of IL-6-type cytokines, including IL-6 and IL-11 [16,101]. IL-6 has potent osteotropic effects: in periodontitis, elevated levels promote osteoclast differentiation and activity, directly contributing to alveolar bone loss [96,101,102].

Bioactive compounds offer a therapeutic strategy to break this self-reinforcing cycle. Many compounds suppress the expression and secretion of TNF-α and IL-1β from GFs. Antcin K, a triterpenoid, inhibits LPS-induced production of IL-1β, IL-6, and other cytokines in human gingival fibroblasts by modulating PI3K/Akt and NF-κB signaling pathways [101]. Other compounds, including resveratrol, kaempferol and veratric acid, exhibit potent anti-inflammatory activities by reducing the production of these key cytokines [20]. By dampening the initial burst of cytokines from GF, these bioactive agents effectively reduce the downstream inflammatory cascade, limit osteoclast activation, and protect periodontal tissues from destruction [101].

Another critical axis in inflammatory processes involves the synthesis of prostaglandins: lipid mediators that promote inflammation, pain, and vasodilation. The enzyme cyclooxygenase-2 (COX-2) is a key catalyst, converting arachidonic acid into prostaglandins, most notably PGE2 [45]. While COX-1 is constitutively expressed and performs housekeeping functions, COX-2 is inducible, with expression dramatically upregulated in response to inflammatory stimuli such as cytokines and bacterial endotoxins, leading to a PGE2 surge, exacerbating the inflammatory state in periodontal tissues [45]. COX-2 expression is regulated by upstream signaling cascades, with the MAPK pathway being crucial, including kinases like ERK, JNK, and p38, activated by inflammatory triggers and activating transcription factors driving COX-2 gene expression [45].

Resveratrol inhibits phosphorylation of ERK, a key MAPK pathway component, blocking ERK activation and preventing downstream signaling leading to increased COX-2 expression, thereby reducing inflammatory prostaglandin synthesis [45]. Synthetic derivatives of pterostilbene, a compound related to resveratrol, are potent and selective COX-2 inhibitors that also modulate MAPK and NF-κB pathways [103]. Other natural compounds like curcumin and EGCG also exert anti-inflammatory effects partly by inhibiting the MAPK pathway and reducing COX-2 activity [45]. By targeting both the COX-2 enzyme and its upstream MAPK regulators, bioactive compounds provide a dual-pronged approach to suppressing a major source of inflammatory mediators in oral diseases [45,103].

Irreversible destruction of periodontal tissues, including collagen-rich periodontal ligament and alveolar bone, is primarily executed by matrix metalloproteinases (MMPs), zinc-dependent endopeptidases degrading all ECM components [104,105]. While MMPs play roles in normal tissue remodeling and wound healing, their expression and activity are pathologically elevated in periodontitis, leading to an imbalance with natural inhibitors, TIMPs, resulting in excessive, uncontrolled ECM degradation [105]. Several MMPs are particularly implicated. MMP-8 (neutrophil collagenase) is the primary culprit, degrading fibrillar type I and III collagens, forming the structural backbone of the gingiva and periodontal ligament [104,105]. MMP-9 (gelatinase B) plays a key role in initiating bone resorption by clearing the protective collagen layer from the bone surface, allowing osteoclast attachment and demineralization [104,105]. Other MMPs, such as MMP-1 and MMP-13, also contribute to this destructive process, with synthesis induced by pro-inflammatory cytokines like IL-1β and TNF-α, activated from latent pro-enzyme form by various factors including reactive oxygen species (ROS) [104].

Modulation of MMP activity is a critical therapeutic strategy to stop periodontitis progression, a concept known as host modulation therapy [106]. Bioactive compounds, both natural and synthetic, show significant promise as MMP inhibitors (MMPIs) [20,21]. Natural compounds such as catechins in green tea extracts, proanthocyanidins from grape seed extracts, and curcumin demonstrate the ability to inhibit MMP activity [56,105]. These compounds act by directly binding enzymes, chelating the essential zinc ion in the active site, or downregulating gene expression [105]. Curcumin inhibits MMP-9 expression and interrupts inflammatory signaling pathways leading to its production [57,105]. On the synthetic side, chemically modified tetracyclines (CMTs), such as sub-antimicrobial dose doxycycline, are well-established MMPIs functioning not through antibiotic effect but by directly inhibiting MMP activity, reducing collagen degradation and preserving periodontal tissues [105,107]. By re-establishing balance between MMPs and TIMPs, bioactive MMPIs serve as powerful adjunctive therapy to mechanical debridement, helping arrest tissue destruction and create an environment conducive to healing and regeneration [104,105].

### 5.3. Antioxidant Action

Oxidative stress—an imbalance between reactive oxygen species (ROS) production and the body’s antioxidant capacity—is a key pathological factor in periodontitis [108]. In inflamed periodontal tissues, activated neutrophils release massive amounts of ROS, contributing to collateral tissue damage [108,109]. Bioactive compounds, particularly polyphenols, counteract oxidative damage through two complementary mechanisms: direct ROS scavenging via hydroxyl groups on aromatic rings that donate electrons to neutralize free radicals [108,110], and activation of the Nrf2/ARE pathway, which upregulates endogenous antioxidant enzymes including superoxide dismutase, catalase, glutathione peroxidase, and heme oxygenase-1 [108]. By directly quenching these damaging molecules, bioactive compounds provide the first line of defense against oxidative injury in the oral cavity [101,108,109]. This dual approach provides more robust protection than direct scavenging alone [110,111]. In the presence of oxidative stress or certain bioactive compounds, Nrf2 is released from Keap1 and translocates to the nucleus. There, it binds the antioxidant response element (ARE) in the promoter region of numerous antioxidant genes, initiating the transcription and synthesis of protective enzymes such as SOD, CAT, GPx, HO-1 and NQO1 [110,112,113,114,115]. These enzymes work together to detoxify ROS and electrophiles, enhancing the cell’s overall antioxidant capacity.

Numerous bioactive compounds are potent activators of the Nrf2 pathway. Carnosic acid, from rosemary, alleviates periodontitis by activating Nrf2/GPX4 signaling axis, which inhibits ferroptosis—iron-dependent cell death driven by lipid peroxidation [113]. BML-111, a lipoxin analog, activates the Nrf2/HO-1 pathway in human periodontal ligament fibroblasts, protecting them from oxidative stress-induced pyroptosis and preserving crucial osteogenic capacity [114]. Other compounds like notopterol, proanthocyanidins, quercetin, and baicalin also exert protective effects partly through activation of this powerful defensive pathway [37]. By amplifying the cell’s intrinsic antioxidant machinery, these compounds provide more robust and lasting protection against oxidative damage compared to direct scavenging alone [37,110,114].

The destructive capacity of oxidative stress is evident in damage inflicted on cellular components, measurable by specific biomarkers. The most common forms are lipid peroxidation and oxidative DNA damage [108,115]. Lipid peroxidation is a chain reaction in which ROS attack lipids, particularly polyunsaturated fatty acids in cell membranes, compromising membrane integrity and leading to loss of function and cell death [108]. Polyphenols are particularly effective in inhibiting lipid peroxidation. In addition to directly scavenging radicals that initiate the process, their ability to chelate transition metals such as iron and copper prevents these metals from catalyzing the formation of highly reactive hydroxyl radicals. The amphiphilic nature of some polyphenols allows integration into cell membranes, where they directly intercept radicals at the potential damage site [115].

Oxidative DNA damage is another serious consequence of oxidative stress, with implications for mutagenesis and carcinogenesis. A primary biomarker for this damage is 8-hydroxy-2′-deoxyguanosine (8-OHdG), formed when a hydroxyl radical attacks the guanine base in the DNA strand [114,115]. Elevated levels of 8-OHdG indicate significant oxidative stress and are associated with various diseases, including oral cancer [114,115]. Polyphenols contribute to reducing 8-OHdG levels primarily by scavenging hydroxyl radicals, causing the damage [115]. By preventing initial oxidative hits to DNA, these compounds preserve genomic integrity. The ability of bioactive compounds to reduce levels of lipid peroxidation products and 8-OHdG serves as a clear indicator of efficacy in mitigating harmful downstream consequences of oxidative stress in the oral environment, contributing to the prevention of diseases ranging from periodontitis to oral cancer [108,115]. Table 1 provides a comprehensive overview of traditional foods with documented effects on oral health, detailing the primary bioactive compounds, target microorganisms, and mechanisms of action.

## 6. Traditional Foods and Salivary Biomarkers

Saliva, a complex biofluid, serves as a critical interface between dietary intake and the oral environment. Its composition reflects local oral health status and systemic physiological changes, making it invaluable for non-invasive diagnostic and prognostic assessment. The analysis of salivary biomarkers provides a real-time snapshot of the biochemical processes occurring within the oral cavity, including changes in pH, levels of oxidative stress, inflammatory responses, and innate immune activity. Understanding how diet modulates these biomarkers is fundamental to elucidating the mechanisms that underlie oral diseases and developing targeted nutritional strategies for prevention and management.

### 6.1. Salivary pH and Buffering Capacity

Maintenance of a neutral pH in the oral cavity is paramount for the preservation of the integrity of dental hard tissue. The inherent buffering system of saliva, primarily driven by bicarbonate, phosphate, and protein components, works continuously to neutralize acids produced by cariogenic bacteria or introduced directly from the diet. A drop in pH below the critical threshold of approximately 5.5 initiates enamel demineralization, the first step in the development of caries and erosion [64,116]. Dietary choices have a profound and immediate impact on this delicate balance. Consumption of acidic beverages is a major challenge to salivary pH homeostasis. Carbonated soft drinks, with intrinsic pH values as low as 2.04 for products, cause a dramatic instantaneous drop in salivary pH [64,117]. A study documented a fall from baseline of 7.18 to 5.65 immediately after consumption, pushing the oral environment into a danger zone of demineralization [64].

Similarly, commercial fruit juices and sports drinks, containing organic acids such as citric acid and often having a pH between 3 and 4, significantly depress both salivary pH and buffering capacity [64,116]. Research involving athletes showed that the consumption of sports drinks during exercise decreased salivary pH by up to 6.6% and buffering capacity by nearly 12%, effects exacerbated by exercise-induced reduction in salivary flow rate [64,116]. In stark contrast, consuming mineral water helps maintain stable salivary parameters, mitigating the risk of dental erosion and caries [116]. The physical form of food also plays a crucial role in cariogenic potential. Liquid sugars are cleared relatively quickly, whereas solid and sticky foods adhere to tooth surfaces for longer periods, extending the duration of acid production and demineralization risk [64].

### 6.2. Oxidative Stress Markers in Saliva

Oxidative stress is a key pathogenic mechanism in chronic inflammatory diseases, including periodontitis. The oral cavity is the site of significant oxidative activity, driven by inflammatory responses to microbial biofilms, activated neutrophils, and dietary factors. Saliva contains a range of biomarkers reflecting the extent of oxidative damage, offering information on the activity of the disease and the efficacy of antioxidant systems. Malondialdehyde (MDA) and 8-hydroxydeoxyguanosine (8-OHdG) are two of the most studied salivary markers of oxidative stress. MDA is a terminal product of lipid peroxidation, indicating damage to cellular membranes, while 8-OHdG is the product of oxidative damage to DNA [64]. Studies consistently demonstrate that salivary concentrations of both MDA and 8-OHdG are significantly elevated in patients with chronic periodontitis compared to healthy individuals [118]. Furthermore, their levels show a strong positive correlation with clinical parameters of the severity of periodontal disease, such as the depth of the probing pocket (PPD), the plaque index and the bleeding on probing (BOP) [64], suggesting that as periodontal inflammation and tissue destruction worsen, the oxidative damage to lipids and DNA increases, the process reflected in saliva.

Complementing the measurement of the damage markers is the assessment of the total antioxidant capacity (TAC). Salivary TAC represents the cumulative action of all antioxidants present in saliva, including enzymes such as SOD and GPx, as well as non-enzymatic molecules such as uric acid, which alone can account for up to 70% of TAC [119]. The relationship between salivary TAC and oral disease is complex. In the periodontitis context, most studies report lower TAC levels in patients compared to healthy controls, suggesting a depleted antioxidant defense system in the face of chronic inflammation [117]. This finding is consistent with observations of an inverse relationship between MDA/8-OHdG levels and activity of antioxidant enzymes like SOD and GPx in periodontitis patients [64]. Conversely, in the case of dental caries, several studies found higher salivary TAC in individuals with caries compared to caries-free subjects [119]. This seemingly paradoxical finding may represent adaptive, compensatory upregulation of the antioxidant system in response to oxidative stress generated by the caries process [118,119].

### 6.3. Inflammatory Cytokines in Saliva

Periodontal disease is a fundamentally inflammatory condition, initiated by dysbiotic microbial biofilm and perpetuated by destructive host immune response. Pro-inflammatory cytokines are key signaling molecules that orchestrate this response, driving the recruitment of immune cells, stimulating tissue-degrading enzymes, and promoting alveolar bone resorption. Saliva serves as a reservoir for these cytokines, released from inflamed periodontal tissues, making them excellent biomarkers for diagnosing and monitoring disease activity. Among the most significant salivary cytokines are IL-1β, TNF-α and IL-6. These three molecules are central players in the inflammatory cascade of periodontitis [42,120]. IL-1β is one of the most well-established biomarkers, with salivary levels significantly elevated in periodontitis patients and showing a strong positive correlation with clinical measures of disease severity, including PPD and clinical attachment level (CAL) [121,122]. It is a potent stimulator of bone resorption and induces secretion of collagenases, directly contributing to the breakdown of periodontal ligament and alveolar bone [121,123].

Similarly, TNF-α levels in saliva are increased in individuals with periodontitis and are associated with the onset and progression of the disease [42,121]. TNF-α not only contributes to bone resorption but also amplifies the inflammatory response by stimulating the production of chemokines, recruiting more immune cells to the infection site [121]. IL-6, another key pro-inflammatory cytokine, demonstrates a strong association with the establishment and severity of periodontitis. Its salivary concentrations correlate positively with plaque index, BOP, PPD, and CAL [122]. Together, IL-1β, TNF-α, and IL-6 are produced by a variety of cells within the periodontium—including macrophages, neutrophils, and fibroblasts—in response to bacterial challenge. They work in concert to activate osteoclastogenesis, a cellular process responsible for alveolar bone loss, the hallmark of advanced periodontitis [42,123]. Measurement of these cytokines in saliva provides a non-invasive means to assess underlying inflammatory burden and has been shown to be useful in monitoring response to periodontal therapy, as their levels typically decrease following successful treatment [121].

### 6.4. Antimicrobial Peptides: Lactoferrin, Lysozyme, and Defensins

Beyond chemical buffering capacity, saliva contains a sophisticated arsenal of antimicrobial peptides (AMPs) and proteins that form a crucial part of the innate immune system, providing the first line of defense against pathogenic microorganisms. These molecules maintain oral homeostasis by inhibiting microbial growth, preventing adhesion to host surfaces, and directly killing potential invaders. Key components include lactoferrin, lysozyme, and defensins. Lactoferrin is an iron-binding glycoprotein with powerful bacteriostatic and bactericidal properties. Its primary mechanism is the sequestration of free iron, an essential nutrient for the growth and proliferation of many bacterial species, including the primary cariogenic bacterium *S. mutans* [64]. By limiting iron availability, lactoferrin effectively starves bacteria. It has also been shown to inhibit the adhesion of *S. mutans* to tooth surfaces and has antiviral and immunomodulatory functions, as well as promoting wound healing [120].

The lysozyme is an enzyme that targets the structural integrity of bacterial cell walls. It hydrolyses the peptidoglycan layer characteristic of many bacteria. This action weakens the cell wall, making the bacterium susceptible to osmotic lysis and death [64,120]. Inclusion of lactoferrin and lysozyme in artificial saliva substitutes has been shown to confer antibacterial effects against *S. mutans*, underscoring their importance in the prevention of caries [64]. Defensins are a family of small cationic peptides with broad-spectrum antimicrobial activity against bacteria, fungi, and viruses. Their positive charge allows binding to negatively charged microbial cell membranes, where they insert and form pores. This disruption of membrane integrity leads to the leakage of essential intracellular contents and ultimately cell death [64,119,120]. In the oral cavity, two main types are present: alpha-defensins, produced primarily by neutrophils and found at sites of inflammation, and beta-defensins (hBDs), expressed by epithelial cells [64,120]. Although hBD-1 is constitutively expressed, the expression of hBD-2 and hBD-3 is inducible, upregulated in response to bacterial products and pro-inflammatory cytokines, providing a responsive defense mechanism [64]. Lower salivary levels of alpha-defensins have been correlated with increased susceptibility to dental caries in children, highlighting their protective role [64,119].

### 6.5. Saliva as Diagnostic Fluid

The ease and non-invasive nature of saliva collection make it an exceptionally attractive fluid for diagnostics, disease monitoring, and research. The biomarkers they contain offer a wealth of information about local oral health and can even reflect the states of systemic disease. For example, a strong correlation between salivary inflammatory cytokines and the clinical parameters of periodontitis suggests their potential as tools for early detection, assessing disease severity without the need for a comprehensive clinical examination, and monitoring treatment effectiveness [121,122]. Similarly, markers of oxidative stress provide information on the underlying pathogenic processes of periodontitis and caries [64,124]. However, translation of these biomarkers into routine clinical practice faces challenges. A significant limitation is the high degree of intraindividual variability observed for many markers. Studies in healthy individuals show that levels of the oxidative stress markers MDA and 8-OHdG can fluctuate significantly throughout the day and day to day [64]. This inherent biological noise means that a single measurement may not be a reliable indicator of an individual’s true baseline or disease status, potentially requiring multiple samples collected over time for accurate assessment. The development of standardized collection and analysis protocols, along with the establishment of robust reference ranges and diagnostic cut-offs, is essential for future clinical utility.

## 7. Clinical and Translational Evidence

Although laboratory studies and biomarker analyses provide mechanistic insights, clinical and translational research are essential to validate the real-world impact of diet on oral health [22]. This body of evidence, derived from human studies, evaluates the effectiveness of specific diet patterns, foods, and nutrients in the prevention and management of oral diseases. Randomized controlled trials (RCTs), systematic reviews, and epidemiological studies have begun to build a strong case for the integration of nutritional counselling into routine dental care, demonstrating that targeted dietary changes can produce measurable improvements in oral health outcomes.

### 7.1. Dietary Interventions and Oral Health Outcomes

A systematic review [125] of RCTs that evaluated dietary interventions delivered in dental care settings found some evidence of effectiveness, although the results were mixed. The review noted that the interventions were more successful in changing behaviors related to fruit, vegetable and alcohol consumption than in reducing sugar intake to prevent dental caries. Evidence for sugar reduction was deemed less conclusive, in part because many studies were multi-interventional, making it difficult to isolate the effect of diet advice alone. This highlights a significant challenge in nutritional research: disentangling the effects of individual dietary components from the broader context of multi-faceted intervention.

Another area of active investigation is the use of functional and nutritional supplements as adjuncts to conventional periodontal therapy. A systematic review of RCTs examined the effects of various nutritional and nutraceutical interventions in conjunction with non-surgical periodontal therapy (NSPT), such as scaling and root planning [126]. The findings were promising for several interventions. Significant positive effects on periodontal parameters, including reductions in the depth of the probing of the pocket and bleeding on probing, were observed for the adjunctive use of vitamin E, chicory extract, certain juice powders and the consumption of green and oolong tea [127]. These substances are rich in antioxidants and anti-inflammatory compounds, likely helping to quell the destructive inflammatory processes of periodontitis. In contrast, other supplements such as lycopene, folate, omega-3 fatty acids, and vitamin D showed more heterogeneous or inconsistent effects, while consumption of kiwifruit did not show a significant benefit at pocket depths [126]. These trials underscore the potential for targeted nutritional support to improve the results of mechanical periodontal treatment, suggesting that the combination of professional cleaning and a supportive anti-inflammatory diet may be more effective than either approach alone.

### 7.2. Fermented Foods and Probiotics in Oral Health

The concept of modulating the oral microbiome for health benefits has gained significant traction, with a growing body of research focused on probiotics—live microorganisms conferring health benefits on the host [128]. Delivered via supplements or fermented foods, probiotics are believed to improve oral health by competing with pathogenic bacteria for adhesion sites and nutrients, producing antimicrobial substances, and modulating the host’s immune response to be less inflammatory [127]. Efficacy of probiotics is highly strain-specific, meaning benefits observed with one bacterial strain cannot be generalized to others.

Clinical trials have explored the use of probiotics for a range of oral conditions. For oral candidiasis, an opportunistic fungal infection, certain strains of lactic acid bacteria, such as *Lacticaseibacillus rhamnosus* and *Lactobacillus acidophilus*, showed promise in reducing oral *Candida* counts, particularly in vulnerable populations like the elderly and denture wearers [128]. In the context of periodontal disease, strains like *Limosilactobacillus reuteri* and *Levilactobacillus brevis* improved gingival health, leading to decreased gum bleeding and reduced levels of pro-inflammatory cytokines in the gingival crevicular fluid [129]. Evidence for dental caries prevention is more tentative. Although some studies report that consumption of products containing probiotic lactobacilli or bifidobacteria can reduce salivary counts of cariogenic mutans streptococci, this effect is not universally observed in all trials [127]. Key consideration is that temporary reductions in salivary bacterial counts do not always translate to a clinically significant reduction in caries development. Furthermore, most probiotic strains do not permanently colonize the oral cavity, suggesting that continuous long-term consumption is necessary to maintain any beneficial effects [127,130,131].

### 7.3. Traditional and Anti-Inflammatory Diets

Beyond single nutrients or probiotics, research has increasingly focused on the holistic impact of entire diet patterns on oral health. Traditional diets, characterized by whole, unprocessed foods and specific anti-inflammatory diets like the Mediterranean diet, have been associated with better oral health outcomes compared to modern Western diets, typically high in refined sugars, saturated fats and processed foods.

The Mediterranean diet, rich in vegetables, fruits, fatty fish, and olive oil, is well-known for systemic anti-inflammatory benefits. Recent clinical trials extended these benefits to oral health. A landmark RCT demonstrated that the 6-week Mediterranean diet intervention significantly reduced gingival inflammation, measured by the gingival index and bleeding on examination, in individuals with gingivitis [61,132]. Remarkably, this improvement occurred even though plaque levels remained unchanged, strongly suggesting the diet’s anti-inflammatory components directly modulated the host’s inflammatory response in gums, independent of bacterial load [61]. Further research using the Mediterranean Diet Adherence Screener (MEDAS) confirmed a significant negative correlation between adherence to diet and periodontal inflammatory parameters, reinforcing the protective effect of this dietary pattern [59].

Similarly, consumption of green tea, a staple in many traditional Asian diets, has been clinically shown to benefit periodontal health. Green tea is rich in polyphenols, particularly EGCG, which possess potent antioxidant, anti-inflammatory, and antimicrobial properties [48]. An RCT found that using green tea supplements as an adjunct to scaling and root planing resulted in significantly greater improvements in clinical parameters and an eight-fold increase in the total antioxidant capacity of gingival crevicular fluid compared to SRP alone [48]. Epidemiological studies support these findings, showing an inverse correlation between daily green tea intake and the severity of periodontal disease in a large population of Japanese men [48,133].

The broader evidence of traditional diets provides a stark contrast to modern dietary habits. Historically, isolated communities consuming diets low in free sugars exhibited extremely low rates of dental caries [134]. Introduction of Westernized diets high in fermentable carbohydrates invariably leads to a rapid increase in the prevalence of caries in these populations. This relationship is one of the strongest findings in dental research. Traditional dietary components such as dairy products (providing calcium and casein), whole foods rich in fiber (which stimulate saliva) and certain nuts are considered cariostatic or protective against caries [135]. Conversely, the high frequency and amount of free sugar and acid consumption in modern diets are the primary drivers of dental caries and erosion, respectively [134,135].

## 8. Challenges and Research Gaps

Despite significant advances in understanding the intricate links between diet, oral microbiome, and oral health, numerous research gaps and methodological challenges remain. Addressing these limitations is crucial for translating scientific findings into effective public health policies and evidence-based clinical recommendations.

One of the primary challenges in biomarker research is the need for standardization. The high intraindividual variability of markers such as MDA and 8-OHdG complicates their use for individual diagnostics [64]. Future research must focus on establishing standardized protocols for saliva collection, processing, and analysis to minimize technical variability. In addition, large-scale longitudinal studies are needed to establish robust reference ranges and clinically significant cut-off values that account for factors such as age, sex, and time of day. The development of multiplex assays that can simultaneously measure a panel of biomarkers (e.g., inflammatory, oxidative, and microbial markers) may provide a more holistic and reliable assessment of oral health status than any single marker alone.

In the realm of dietary interventions, there is a clear need for more high-quality, long-term RCTs. Many existing studies are short-duration or suffer from methodological weaknesses, such as a lack of blinding or difficulty in isolating the effects of a specific dietary component being tested [22,83,132]. Future trials should be designed with greater methodological rigor and conducted in real-world settings, such as general dental practices, to assess the feasibility and effectiveness of nutritional counselling as a routine part of dental care. There is also a specific need for studies focusing on dietary interventions for the prevention of dental erosion, an area currently under-researched [22].

The promising field of probiotics requires further investigation to move from general concepts to specific evidence-based recommendations. The specific nature of probiotic effects means that future clinical trials must clearly identify strains, dosages, and delivery vehicles used [127,130]. Research should aim to identify optimal probiotic strains for specific oral conditions, whether it is *L. rhamnosus* GG for candidiasis or *L. reuteri* for gingivitis, and determine the most effective administration methods to ensure sufficient colonization and sustained biological activity. Critical questions regarding optimal treatment duration, long-term safety, and potential interactions with the resident oral microbiota also warrant systematic investigation [131].

A critical gap is the limited understanding of the bioavailability and pharmacokinetics of bioactive compounds in the oral environment. Although compounds such as polyphenols demonstrate potent effects in vitro, their efficacy in vivo can be limited by rapid metabolism, poor absorption, and short residence time in the oral cavity. Moreover, a substantial proportion of the mechanistic evidence reviewed derives from in vitro assays and animal models, which may not fully recapitulate the complexity of the human oral environment. For many individual compounds—such as antcin K, carnosic acid, and BML-111—the available evidence is limited to cell culture experiments and preclinical models, and human clinical data are currently absent. Furthermore, much of the mechanistic discussion draws upon comprehensive reviews rather than primary studies, making it difficult to assign a single evidence level to each claim. Translation of these preclinical findings into evidence-based clinical recommendations will require well-designed, adequately powered randomized controlled trials that evaluate the specific oral health outcomes of individual bioactive compounds in diverse human populations.

The development of advanced delivery systems, such as nanoencapsulation, mucoadhesive gels, slow-release films, and incorporation into oral care products, may significantly improve therapeutic efficacy [21,90]. Such delivery technologies could provide sustained release and prolonged contact time with oral tissues, improving clinical outcomes. Future research must adopt more comprehensive, multilevel approaches that acknowledge the complex interplay between diet, genetics, microbiome, behavior, and socioeconomic factors [22]. The focus is shifting from individual nutrients to whole dietary patterns, such as the Mediterranean diet, which may have synergistic effects greater than the sum of individual components. Integrating advanced “omics” technologies (e.g., metabolomics, proteomics, metagenomics) will allow a deeper understanding of the molecular mechanisms by which diet influences the oral environment. This will pave the way for personalized nutrition strategies, where dietary recommendations can be tailored to the individual’s unique genetic and microbial profile to optimize their oral and systemic health.

## 9. Conclusions

This comprehensive review establishes that traditional foods from diverse geographic regions contain bioactive compounds with scientifically supported properties relevant to the prevention and management of oral diseases. The oral microbiome, comprising over 700 bacterial species along with fungi, archaea, and viruses, functions as a critical gatekeeper of both oral and systemic health. Dysbiotic shifts in this microbial ecosystem drive the pathogenesis of dental caries and periodontitis through biofilm formation, acid production, chronic inflammation, and tissue destruction, with consequences extending beyond the oral cavity to cardiovascular disease, diabetes mellitus, adverse pregnancy outcomes, and other systemic conditions.

Traditional dietary components offer a multi-targeted therapeutic approach to these complex conditions. Compounds such as EGCG from green tea, nattokinase from fermented soy, curcumin from turmeric, proanthocyanidins from cranberries, and essential oils from Mediterranean herbs demonstrate potent antimicrobial activity against key oral pathogens. These compounds employ sophisticated anti-virulence strategies, inhibiting biofilm formation, disrupting quorum-sensing systems, and compromising bacterial membrane integrity. Their anti-inflammatory properties are equally notable, modulating key signaling pathways including NF-κB and MAPK while suppressing pro-inflammatory cytokines and matrix metalloproteinases. Additionally, these compounds combat oxidative stress through direct free radical scavenging and activation of endogenous antioxidant defenses.

Clinical evidence supports the efficacy of traditional foods and anti-inflammatory dietary patterns in modulating salivary biomarkers, reducing gingival inflammation, and enhancing periodontal treatment outcomes. However, significant research gaps remain, including high intraindividual variability in salivary biomarkers, an incomplete understanding of bioactive compound bioavailability, and a critical need for rigorous long-term RCTs with oral health endpoints.

In conclusion, traditional foods represent a promising yet underutilized tool for promoting oral and systemic health. Their integration into preventive strategies may offer low-cost, culturally acceptable options for reducing the burden of oral diseases. Future research combining mechanistic studies, biomarker validation, clinical trials, and implementation science is essential to translate this knowledge into tangible public health improvements. As the evidence base continues to grow, nutritional counseling focusing on traditional, anti-inflammatory dietary patterns should become a standard component of comprehensive oral health care.

## Figures and Tables

**Table 1:** Bioactive Compounds in Traditional Foods: Antimicrobial Mechanisms and Effects on Periodontal Health.

Traditional Food	Primary Bioactive Compound(s)	Target Microorganisms	Mechanism of Action	Key Findings	Reference
Green tea	Epigallocatechin-3-gallate	*S. mutans*, *P. gingivalis*, *F. nucleatum*	Inhibition of glucosyltransferases; bacterial membrane disruption; reduced acid production	8-fold increase in GCF antioxidant capacity when combined with SRP	[46,52]
Natto (fermented soybean)	Nattokinase	*S. mutans*, *S. sobrinus*	Degradation of water-insoluble glucan matrix; inhibition of EPS synthesis	Specific inhibition of sucrose-dependent biofilm formation	[54,55]
Turmeric	Curcumin	*S. mutans*, host inflammatory cells	Downregulation of the *atpH* gene; inhibition of NF-κB and NLRP3 pathways; reduction in IL-1β, IL-6, TNF-α	Reduced plaque and gingival indices comparable to chlorhexidine	[57]
Extra virgin olive oil	Hydroxytyrosol	Oxidative stress mediators	Free radical scavenging; protection of LDL from oxidation	Mediterranean diet adherence associated with reduced periodontal inflammation	[58,62]
Oregano and thyme	Carvacrol, thymol	Gram-positive and Gram-negative oral pathogens	Disruption of bacterial membrane integrity; increased permeability; cell lysis	Broad-spectrum antimicrobial activity against oral bacteria	[86]
Cranberry	A-type proanthocyanidins	*S. mutans*, *P. gingivalis*	Inhibition of bacterial adhesion and glucosyltransferases; reduction in IL-8 and NF-κB activation	Anti-adhesion effects; attenuation of *P. gingivalis* virulence factors	[72]
Cocoa	Flavonoids, polyphenols	*S. mutans*, *F. nucleatum*	Inhibition of bacterial growth, adherence, and glycosyltransferase activity	Cocoa mouthwash reduced salivary *S. mutans* counts and biofilm accumulation	[17]
Propolis	Flavonoids, phenolic acids	*S. mutans*, *P. gingivalis*, *F. nucleatum*	Disruption of microbial membranes; growth inhibition	Reduction in plaque accumulation, gingival inflammation, and bacterial fermentation	[67,69]
Cardamom	Essential oils, phenolic compounds	*A. actinomycetemcomitans*, *P. gingivalis*	Antibacterial activity; biofilm inhibition; reduction in IL-1β and TNF-α via NF-κB inhibition	Dual antimicrobial and anti-inflammatory action demonstrated	[70]
Clove	Eugenol	Cariogenic bacteria	Antiseptic and antibacterial activity; anti-inflammatory effects	Validated efficacy against the oral microbiota	[87]
Cinnamon	Cinnamaldehyde	*S. mutans*	Inhibition of the *vicR* gene; downregulation of *gtfB*/C/D; quorum sensing disruption	Improved periodontal parameters with cinnamon-based interventions	[88]
Yerba mate	Chlorogenic acid, polyphenols, saponins	Oral microbiome	Antioxidant and anti-inflammatory activity	Modulation of oral microbiome composition; reduction in oxidative stress	[21,46,89]

Abbreviations: EPS, extracellular polysaccharides; GCF, gingival crevicular fluid; IL, interleukin; LDL, low-density lipoprotein; NF-κB, nuclear factor kappa-B; NLRP3, NOD-like receptor protein 3; SRP, scaling and root planing; TNF-α, tumor necrosis factor-alpha. Note: Bacterial species are abbreviated after first mention: *S. mutans* (*Streptococcus mutans*); *P. gingivalis* (*Porphyromonas gingivalis*); *F. nucleatum* (*Fusobacterium nucleatum*); *S. sobrinus* (*Streptococcus sobrinus*); *A. actinomycetemcomitans* (*Aggregatibacter actinomycetemcomitans*).

## Data Availability

No new data were created or analyzed in this study. Data sharing is not applicable to this article.

## References

[1] Rajasekaran J.J., Krishnamurthy H.K., Bosco J., Jayaraman V., Krishna K., Wang T., Bei K., Rajasekaran D., Yenn (Steven) P., Thirunavukkarasu K. (2024). Oral Microbiome: A Review of Its Impact on Oral and Systemic Health. Microorganisms.

[2] Tian S., Ding T., Li H. (2024). Oral microbiome in human health and diseases. mLife.

[3] Armas-Vega A., Parise-Vasco J.M., Díaz-Segovia M.C., Arroyo-Bonilla D.A., Cabrera-Dávila M.J., Zambrano-Bonilla M.C., González-Arias D., Pérez-Astudillo S., Recalde-Reyes M., Cabrera-Dávila S.P. (2023). Prevalence of Dental Caries in Schoolchildren from the Galapagos Islands: ESSO-Gal Cohort Report. Int. J. Dent..

[4] Caselli E., Fabbri C., D’Accolti M., Soffritti I., Bassi C., Mazzacane S., Franchi M. (2020). Defining the oral microbiome by whole-genome sequencing and resistome analysis: The complexity of the healthy picture. BMC Microbiol..

[5] Kilian M., Chapple I.L.C., Hannig M., Marsh P.D., Meuric V., Pedersen A.M.L., Tonetti M.S., Wade W.G., Zaura E. (2016). The oral microbiome—An update for oral healthcare professionals. Br. Dent. J..

[6] Marsh P.D. (1994). Microbial Ecology of Dental Plaque and its Significance in Health and Disease. Adv. Dent. Res..

[7] Marsh P.D. (2003). Are dental diseases examples of ecological catastrophes?. Microbiology.

[8] Hajishengallis G., Lamont R.J. (2014). Breaking bad: Manipulation of the host response by *Porphyromonas gingivalis*. Eur. J. Immunol..

[9] Herrera D., Sanz M., Shapira L., Brotons C., Chapple I., Frese T., Graziani F., Hobbs F.D.R., Huck O., O’Connor C. (2023). Association between periodontal diseases and cardiovascular diseases, diabetes and respiratory diseases: Consensus report of the Joint Workshop by the European Federation of Periodontology (EFP) and the European arm of the World Organization of Family Doctors (WONCA Europe). J. Clin. Periodontol..

[10] Baker J.L., Mark Welch J.L., Kauffman K.M., McLean J.S., He X. (2024). The oral microbiome: Diversity, biogeography and human health. Nat. Rev. Microbiol..

[11] Chopra A., Franco-Duarte R., Rajagopal A., Choowong P., Soares P., Rito T., Montazeri H., Fernandes T. (2024). Exploring the presence of oral bacteria in non-oral sites of patients with cardiovascular diseases using whole metagenomic data. Sci. Rep..

[12] Lund Håheim A.L. (2024). Oral anaerobe bacteria—A common risk for cardiovascular disease and mortality and some forms of cancer?. Front. Oral Health.

[13] Leng Y., Hu Q., Ling Q., Yao X., Liu M., Chen J., Yan Z. (2023). Periodontal disease is associated with the risk of cardiovascular disease independent of sex: A meta-analysis. Front. Cardiovasc. Med..

[14] Guan H., Zhao S., Tan Y., Fang X., Zhang Y., Zhang Y., Wang S., Li H., Zhang L., Wang Z. (2024). Microbiomic insights into the oral microbiome’s role in type 2 diabetes mellitus: Standardizing approaches for future advancements. Front. Endocrinol..

[15] Lyu X., Xu X., Shen S., Qin F. (2024). Genetics causal analysis of oral microbiome on type 2 diabetes in East Asian populations: A bidirectional two-sample Mendelian randomized study. Front. Endocrinol..

[16] Reytor-González C., Parise-Vasco J.M., González N., Simancas-Racines A., Zambrano-Villacres R., Zambrano A.K., Simancas-Racines D. (2024). Obesity and periodontitis: A comprehensive review of their interconnected pathophysiology and clinical implications. Front. Nutr..

[17] Fideles S.O.M., de Cássia Ortiz A., Reis C.H.B., Buchaim D.V., Buchaim R.L. (2023). Biological Properties and Antimicrobial Potential of Cocoa and Its Effects on Systemic and Oral Health. Nutrients.

[18] Delaire L., Courtay A., Humblot J., Aubertin-Leheudre M., Mourey F., Racine A.N., Israel F., Bonnefoy M. (2023). Implementation and Core Components of a Multimodal Program including Exercise and Nutrition in Prevention and Treatment of Frailty in Community-Dwelling Older Adults: A Narrative Review. Nutrients.

[19] Etebarian A., Alhouei B., Mohammadi-Nasrabadi F., Esfarjani F. (2024). Propolis as a functional food and promising agent for oral health and microbiota balance: A review study. Food Sci. Nutr..

[20] Hashim N.T., Babiker R., Rahman M.M., Mohamed R., Priya S.P., Chaitanya N.C., Eltayeb M.M., Gismalla B.G., Ali R.W. (2024). Natural Bioactive Compounds in the Management of Periodontal Diseases: A Comprehensive Review. Molecules.

[21] Kashi M., Varseh M., Hariri Y., Chegini Z., Shariati A. (2025). Natural compounds: New therapeutic approach for inhibition of *Streptococcus mutans* and dental caries. Front. Pharmacol..

[22] Chamut S., Alhassan M., Hameedaldeen A., Kaplish S., Yang A.H., Wade C.G., Kim S.H., Lee C., Park J.B. (2024). Every bite counts to achieve oral health: A scoping review on diet and oral health preventive practices. Int. J. Equity Health.

[23] Sedghi L., Byron C., Jennings R., Chlipala G.E., Green S.J., Silo-Suh L. (2019). Effect of Dietary Fiber on the Composition of the Murine Dental Microbiome. Dent. J..

[24] Santonocito S., Polizzi A., Palazzo G., Indelicato F., Isola G. (2021). Dietary Factors Affecting the Prevalence and Impact of Periodontal Disease. Clin. Cosmet. Investig. Dent..

[25] Glavin C., Gartshore J., Jackson G., Bonsor S. (2025). Does adopting a healthy diet improve periodontal parameters in patients susceptible to periodontal disease? A systematic review. Evid. Based Dent..

[26] Lin Y., Liang X., Li Z., Gong T., Ren B., Li Y., He T., Zhou X. (2024). Omics for deciphering oral microecology. Int. J. Oral Sci..

[27] Kunath B.J., De Rudder C., Laczny C.C., Letellier E., Wilmes P. (2024). The oral–gut microbiome axis in health and disease. Nat. Rev. Microbiol..

[28] Abdulkareem A.A., Al-Taweel F.B., Al-Sharqi A.J.B., Gul S.S., Sha A., Chapple I.L.C. (2023). Current concepts in the pathogenesis of periodontitis: From symbiosis to dysbiosis. J. Oral Microbiol..

[29] Damoczi J., Knoops A., Martou M.-S., Jaumaux F., Gabant P., Mahillon J., Van der Henst C. (2024). Uncovering the arsenal of class II bacteriocins in salivarius streptococci. Commun. Biol..

[30] Trueb L., Lepori M., Duplain H., Scherrer U., Sartori C. (2012). Nitric oxide mediates the blood pressure response to mental stress in humans. Swiss Med. Wkly..

[31] Cui Z., Wang P., Gao W. (2025). Microbial dysbiosis in periodontitis and peri-implantitis: Pathogenesis, immune responses, and therapeutic. Front. Cell. Infect. Microbiol..

[32] Zhu Y., Wang Y., Zhang S., Li J., Li X., Ying Y., Zhou X., Chen Y., Xu X. (2023). Association of polymicrobial interactions with dental caries development and prevention. Front. Microbiol..

[33] Zhang Q., Ma Q., Wang Y., Wu H., Zou J. (2021). Molecular mechanisms of inhibiting glucosyltransferases for biofilm formation in *Streptococcus mutans*. Int. J. Oral Sci..

[34] Fitri D.K., Tuygunov N., Wan Harun W.H.A., Purwasena I.A., Cahyanto A., Zakaria M.N. (2025). Key virulence genes associated with *Streptococcus mutans* biofilm formation: A systematic review. Front. Oral Health.

[35] Tossetta G., Fantone S., Olivieri F., Mazzucchelli R., Togni L., Santarelli A., Marzioni D. (2025). Effect of natural compounds on NRF2/KEAP1 signaling in periodontitis: A potential use to prevent age-related disorders. Mol. Biol. Rep..

[36] Cox A.J., West N.P., Cripps A.W. (2015). Obesity, inflammation, and the gut microbiota. Lancet Diabetes Endocrinol..

[37] Niwano Y., Shishido S., Shirato M., Kohzaki H., Nakamura K. (2025). Therapeutic Potential of Proanthocyanidins in Dentistry: A Focus on Periodontal Disease and on Dental Implants in Osteoporotic Patients. Antioxidants.

[38] Guo X., Dai S., Lou J., Ma X., Hu X., Tu L., Zhu Y., Zhang Y., Chen T., Xie G. (2023). Distribution characteristics of oral microbiota and its relationship with intestinal microbiota in patients with type 2 diabetes mellitus. Front. Endocrinol..

[39] AlSharief M., Alabdurubalnabi E. (2023). Periodontal Pathogens and Adverse Pregnancy Outcomes: A Narrative Review. Life.

[40] Nazir M.A. (2017). Prevalence of periodontal disease, its association with systemic diseases and prevention. Int. J. Health Sci..

[41] Harrandah A.M. (2025). The Oral–Gut–Systemic Axis: Emerging Insights into Periodontitis, Microbiota Dysbiosis, and Systemic Disease Interplay. Diagnostics.

[42] Neurath N., Kesting M. (2024). Cytokines in gingivitis and periodontitis: From pathogenesis to therapeutic targets. Front. Immunol..

[43] Şenel S. (2021). An Overview of Physical, Microbiological and Immune Barriers of Oral Mucosa. Int. J. Mol. Sci..

[44] Bradley E., Haran J. (2024). The human gut microbiome and aging. Gut Microbes.

[45] Xu Q., Wang W., Li Y., Cui J., Zhu M., Liu Y., Zhang H., Chen Y., Zhao L. (2025). The oral-gut microbiota axis: A link in cardiometabolic diseases. npj Biofilms Microbiomes.

[46] Arzani V., Soleimani M., Fritsch T., Jacob U.M., Calabrese V., Arzani A. (2025). Plant polyphenols, terpenes, and terpenoids in oral health. Open Med..

[47] Huang Y.-Q., Lu X., Min H., Wu Q.-Q., Shi X.-T., Bian K.-Q., Zou X.-P. (2016). Green tea and liver cancer risk: A meta-analysis of prospective cohort studies in Asian populations. Nutrition.

[48] Chopra A., Thomas B.S., Sivaraman K., Prasad H.K., Kamath S.U. (2016). Green Tea Intake as an Adjunct to Mechanical Periodontal Therapy for the Management of Mild to Moderate Chronic Periodontitis: A Randomized Controlled Clinical Trial. Oral Health Prev. Dent..

[49] Gerits E., Verstraeten N., Michiels J. (2017). New approaches to combat *Porphyromonas gingivalis* biofilms. J. Oral Microbiol..

[50] Yuvaraja M., Reddy N.R., Kumar P.M., Ravi K., Alqahtani N. (2016). Thermoreversible gel for intrapocket delivery of green tea catechin as a local drug delivery system: An original research. J. Adv. Pharm. Technol. Res..

[51] Song P., Hao Y., Lin D., Jin Y., Lin J. (2024). Evaluation of the antibacterial effect of Epigallocatechin gallate on the major pathogens of canine periodontal disease and therapeutic effects on periodontal disease mice. Front. Microbiol..

[52] Wang Y., Zeng J., Yuan Q., Luan Q. (2021). Efficacy of (−)-epigallocatechin gallate delivered by a new-type scaler tip during scaling and root planing on chronic periodontitis: A split-mouth, randomized clinical trial. BMC Oral Health.

[53] Leñini C., Rodriguez Ayala F., Goñi A.J., Rateni L., Nakamura A., Grau R.R. (2023). Probiotic properties of *Bacillus subtilis* DG101 isolated from the traditional Japanese fermented food nattō. Front. Microbiol..

[54] Kimijima M., Narisawa N., Hori E., Mandokoro K., Ito T., Ota Y., Ikeda T., Senpuku H. (2024). Nattokinase, a Subtilisin-like Alkaline-Serine Protease, Reduces Mutacin Activity by Inactivating the Competence-Stimulating Peptide in *Streptococcus mutans*. Pathogens.

[55] Zhang J., Bilal M., Liu S., Zhang J., Lu H., Luo H., Zhu Y., Stålbrand H., Ni H. (2020). Isolation, Identification and Antimicrobial Evaluation of Bactericides Secreting *Bacillus subtilis* Natto as a Biocontrol Agent. Processes.

[56] Reddy M.S., Ramachandra S.S., Shetty S.R., Khazi S.S., ur Rahman Tippu M.S., Narayanan L.A., Balasubramanian S. (2025). Analgesic Efficacy of Phytotherapeutic Agents in Dental Pain Management: A Systematic Review. Int. J. Dent..

[57] Al-Maweri S.A., Alhajj M.N., Deshisha E.A., Alshafei A.K., Ahmed A.I., Almudayfi N.O., Al-Soneidar W.A., Alsharani A. (2022). Curcumin mouthwashes versus chlorhexidine in controlling plaque and gingivitis: A systematic review and meta-analysis. Int. J. Dent. Hyg..

[58] Ben Hassena A., Abidi J., Miled N., Kulinowski Ł., Skalicka-Woźniak K., Bouaziz M. (2025). New Insights into the Antibacterial Activity of Hydroxytyrosol Extracted from Olive Leaves: Molecular Docking Simulations of its Antibacterial Mechanisms. Chem. Biodivers..

[59] Bartha V., Exner L., Meyer A.-L., Basrai M., Schweikert D., Adolph M., Bischoff S.C. (2022). How to Measure Adherence to a Mediterranean Diet in Dental Studies: Is a Short Adherence Screener Enough? A Comparative Analysis. Nutrients.

[60] Ussia S., Ritorto G., Mollace R., Serra M., Tavernese A., Altomare C., Gliozzi M., Musolino V., Carresi C., Maiuolo J. (2025). Exploring the Benefits of Extra Virgin Olive Oil on Cardiovascular Health Enhancement and Disease Prevention: A Systematic Review. Nutrients.

[61] Joshi C., Bapat R., Anderson W., Dawson D., Cherukara G., Hijazi K. (2021). Serum antibody response against periodontal bacteria and coronary heart disease: Systematic review and meta-analysis. J. Clin. Periodontol..

[62] Bender C., Candi I., Rogel E. (2023). Efficacy of Hydroxytyrosol-Rich Food Supplements on Reducing Lipid Oxidation in Humans. Int. J. Mol. Sci..

[63] D’Angelo C., Franceschelli S., Quiles J.L., Speranza L. (2020). Wide Biological Role of Hydroxytyrosol: Possible Therapeutic and Preventive Properties in Cardiovascular Diseases. Cells.

[64] Nguyen T.T., Ngo L.Q., Promsudthi A., Surarit R. (2017). Salivary oxidative stress biomarkers in chronic periodontitis and acute coronary syndrome. Clin. Oral Investig..

[65] Mráz P., Kopecký M., Hasoňová L., Hoštičková I., Vaníčková A., Perná K., Kašparová M., Hrabák J. (2025). Antibacterial Activity and Chemical Composition of Popular Plant Essential Oils and Their Positive Interactions in Combination. Molecules.

[66] Jean-Marie E., Bereau D., Robinson J.-C. (2021). Benefits of Polyphenols and Methylxanthines from Cocoa Beans on Dietary Metabolic Disorders. Foods.

[67] Botelho M.P.J., da Silva A., Antônio Ferreira F da C., Capel L.M.M. (2017). Avaliação in vitro da Atividade Antimicrobiana de Extrato Alcoólico de Própolis Comparado à Solução de Clorexidina 0, 12%. J. Health Sci..

[68] Ayoob A., Janakiram C., Priya M.K. (2024). Spice-Based Herbal Oral Care Products as an Intervention in the Periodontal Diseases: A Systematic Scoping Review. J. Herb. Med..

[69] Deng Y., Liu D., Dissanayake I., Jaye K., Bhuyan D.J., Low M., Li C.G. (2025). Propolis as a functional food ingredient: Modulation of gut microbiota and implications for chronic disease management. Food Res. Int..

[70] Jawdekar A., Saraf T., Tirupathi S., Thribhuvanan L., Deolikar S. (2024). Comparative Evaluation of the Antimicrobial Efficacy of *Elettaria cardamomum* (0.5%) Mouthwash, *Camellia sinensis* (0.5%) Mouthwash, and 0.12% Chlorhexidine Gluconate Mouthwash against *Streptococcus mutans*: An In Vitro Study. Int. J. Clin. Pediatr. Dent..

[71] Pellerin G., Bazinet L., Grenier D. (2021). Deacidification of Cranberry Juice Reduces Its Antibacterial Properties against Oral Streptococci but Preserves Barrier Function and Attenuates the Inflammatory Response of Oral Epithelial Cells. Foods.

[72] Feghali K., Feldman M., La V.D., Santos J., Grenier D. (2012). Cranberry Proanthocyanidins: Natural Weapons against Periodontal Diseases. J. Agric. Food Chem..

[73] La V.D., Howell A.B., Grenier D. (2010). Anti-Porphyromonas gingivalis and Anti-Inflammatory Activities of A-Type Cranberry Proanthocyanidins. Antimicrob. Agents Chemother..

[74] Philip N., Leishman S.J., Bandara H.M.H.N., Healey D.L., Walsh L.J. (2020). Randomized Controlled Study to Evaluate Microbial Ecological Effects of CPP-ACP and Cranberry on Dental Plaque. JDR Clin. Transl. Res..

[75] Chopra A., Avishikta B., Puzhankara L. (2020). Are probiotics an effective alternative to conventional antimicrobials agents for the management of periodontal diseases: A systematic review and meta-analysis. PROSPERO.

[76] Di Stasi M., Kaboudari A., Simone M., Vacchina V., Braca A., De Leo M., Bucchini A. (2025). Traditional Middle Eastern spice blends (Baharat): Antimicrobial activity, metabolomic profile, and trace element analysis. Phytochem. Lett..

[77] Talib W.H., AlHur M.J., Al Naimat S., Ahmad R.E., Al-Yasari A.H., Al-Dalaeen A., Mahmod A.I. (2022). Anticancer Effect of Spices Used in Mediterranean Diet: Preventive and Therapeutic Potentials. Front. Nutr..

[78] Souissi M., Azelmat J., Chaieb K., Grenier D. (2020). Antibacterial and anti-inflammatory activities of cardamom (*Elettaria cardamomum*) extracts: Potential therapeutic benefits for periodontal infections. Anaerobe.

[79] Karygianni L., Cecere M., Skaltsounis A.L., Argyropoulou A., Hellwig E., Aligiannis N., Wittmer A., Al-Ahmad A. (2014). High-Level Antimicrobial Efficacy of Representative Mediterranean Natural Plant Extracts against Oral Microorganisms. Biomed Res. Int..

[80] Beresescu G., Bereczki-Temistocle D.L., Beresescu L., Ormenisan A., Monea A., Razvan-Marius I. (2025). Effectiveness of an Essential Oil Mouthwash on Halitosis in Obese Patients with Periodontitis: A Short-Term Clinical Evaluation. J. Clin. Med..

[81] Alsulaimani A.F., Alfehaid K.W., Alhabash K.M., AlShehri M.A., Alanzi T.S., Alsalem R.S., Aljafar A.S. (2024). Effect of Herbal Medication and Supplements on Oral Health. J. Healthc. Sci..

[82] Yanakiev S. (2020). Effects of Cinnamon (*Cinnamomum* spp.) in Dentistry: A Review. Molecules.

[83] Chatzopoulos G.S., Karakostas P., Kavakloglou S., Assimopoulou A., Barmpalexis P., Tsalikis L. (2022). Clinical Effectiveness of Herbal Oral Care Products in Periodontitis Patients: A Systematic Review. Int. J. Environ. Res. Public Health.

[84] Malcangi G., Inchingolo A.M., Casamassima L., Trilli I., Ferrante L., Inchingolo F., Di Venere D., Palermo A., Inchingolo A.D., Dipalma G. (2025). Effectiveness of Herbal Medicines with Anti-Inflammatory, Antimicrobial, and Antioxidant Properties in Improving Oral Health and Treating Gingivitis and Periodontitis: A Systematic Review. Nutrients.

[85] Xu X., Zhou X.D., Wu C.D. (2011). The Tea Catechin Epigallocatechin Gallate Suppresses Cariogenic Virulence Factors of *Streptococcus mutans*. Antimicrob. Agents Chemother..

[86] Kachur K., Suntres Z. (2020). The antibacterial properties of phenolic isomers, carvacrol and thymol. Crit. Rev. Food Sci. Nutr..

[87] Xu J.-S., Li Y., Cao X., Cui Y. (2013). The effect of eugenol on the cariogenic properties of *Streptococcus mutans* and dental caries development in rats. Exp. Ther. Med..

[88] He Z., Huang Z., Jiang W., Zhou W. (2019). Antimicrobial Activity of Cinnamaldehyde on *Streptococcus mutans* Biofilms. Front. Microbiol..

[89] Klotz S.A., Bradley N., Lipke P.N. (2022). Blocking Serum Amyloid-P Component from Binding to Macrophages and Augmenting Fungal Functional Amyloid Increases Macrophage Phagocytosis of *Candida albicans*. Pathogens.

[90] Gao Z., Chen X., Wang C., Song J., Xu J., Liu X., Qian Y., Zhong W. (2024). New strategies and mechanisms for targeting *Streptococcus mutans* biofilm formation to prevent dental caries: A review. Microbiol. Res..

[91] Merra G., Noce A., Marrone G., Cintoni M., Tarsitano M.G., Capacci A., De Lorenzo A. (2020). Influence of Mediterranean Diet on Human Gut Microbiota. Nutrients.

[92] Parida S., Sharma D. (2019). The Microbiome-Estrogen Connection and Breast Cancer Risk. Cells.

[93] Guo Y., Li Z., Chen F., Chai Y. (2023). Polyphenols in Oral Health: Homeostasis Maintenance, Disease Prevention, and Therapeutic Applications. Nutrients.

[94] Behzadnia A., Moosavi-Nasab M., Oliyaei N. (2024). Anti-biofilm activity of marine algae-derived bioactive compounds. Front. Microbiol..

[95] Li Y., Xing Z., Wang S., Wang Y., Wang Z., Dong L. (2023). Disruption of biofilms in periodontal disease through the induction of phase transition by cationic dextrans. Acta Biomater..

[96] Murugaiyan V., Utreja S., Hovey K.M., Sun Y., LaMonte M.J., Wactawski-Wende J., Buck M.J. (2024). Defining *Porphyromonas gingivalis* strains associated with periodontal disease. Sci. Rep..

[97] Kim D., Han S.K., Lee K., Kim I., Kong J., Kim S. (2019). Evolutionary coupling analysis identifies the impact of disease-associated variants at less-conserved sites. Nucleic Acids Res..

[98] Zhang S., Lin Z.-N., Yang C.-F., Shi X., Ong C.-N., Shen H.-M. (2004). Suppressed NF-κB and sustained JNK activation contribute to the sensitization effect of parthenolide to TNF-α-induced apoptosis in human cancer cells. Carcinogenesis.

[99] Zhao Y., Wu J., Liu X., Chen X., Wang J. (2025). Decoding nature: Multi-target anti-inflammatory mechanisms of natural products in the TLR4/NF-κB pathway. Front. Pharmacol..

[100] Xie L., Wang Y., Gong Y. (2025). Albiflorin improves diabetic retinopathy by mitigating oxidative stress and inflammation via the TLR-4/NF-kB signaling pathway. Toxicol. Res..

[101] Wu Y.-H., Kuo Y.-H., Lin Y.-Y., Shieh T.-M., Chang T.-C., Chang A.-C., Hsia S.-M. (2025). Antcin K suppresses proinflammatory cytokines expression via the PI3K, Akt and NF-κB pathways in human gingival fibroblasts: Implications for periodontitis treatment. Cell Death Discov..

[102] Dorrington M.G., Fraser I.D.C. (2019). NF-κB Signaling in Macrophages: Dynamics, Crosstalk, and Signal Integration. Front. Immunol..

[103] Liu Y., You Y., Lu J., Chen X., Yang Z. (2020). Recent Advances in Synthesis, Bioactivity, and Pharmacokinetics of Pterostilbene, an Important Analog of Resveratrol. Molecules.

[104] Scanu M., Del Chierico F., Marsiglia R., Toto F., Guerrera S., Valeri G., Vicari S., Putignani L. (2024). Correction of Batch Effect in Gut Microbiota Profiling of ASD Cohorts from Different Geographical Origins. Biomedicines.

[105] Ferreira do Couto M.L., Fonseca S., Pozza D.H. (2024). Pharmacogenetic Approaches in Personalized Medicine for Postoperative Pain Management. Biomedicines.

[106] Checchi V., Maravic T., Bellini P., Generali L., Consolo U., Breschi L., Mazzoni A. (2020). The Role of Matrix Metalloproteinases in Periodontal Disease. Int. J. Environ. Res. Public Health.

[107] Griffin M.O., Ceballos G., Villarreal F.J. (2011). Tetracycline compounds with non-antimicrobial organ protective properties: Possible mechanisms of action. Pharmacol. Res..

[108] Vo T.T.T., Chu P.-M., Tuan V.P., Te J.S.-L., Lee I.-T. (2020). The Promising Role of Antioxidant Phytochemicals in the Prevention and Treatment of Periodontal Disease via the Inhibition of Oxidative Stress Pathways: Updated Insights. Antioxidants.

[109] Juan C.A., Pérez de la Lastra J.M., Plou F.J., Pérez-Lebeña E. (2021). The Chemistry of Reactive Oxygen Species (ROS) Revisited: Outlining Their Role in Biological Macromolecules (DNA, Lipids and Proteins) and Induced Pathologies. Int. J. Mol. Sci..

[110] Javed H.U., Liu R., Li C., Zhong S., Lai J., Hasan M., Zhao M. (2023). Preparation of Vanillin-Taurine Antioxidant Compound, Characterization, and Evaluation for Improving the Post-Harvest Quality of Litchi. Antioxidants.

[111] Velichkova M., Hasson T. (2005). Keap1 Regulates the Oxidation-Sensitive Shuttling of Nrf2 into and out of the Nucleus via a Crm1-Dependent Nuclear Export Mechanism. Mol. Cell. Biol..

[112] Satoh T., Okamoto S.-i., Cui J., Watanabe Y., Furuta K., Suzuki M., Tohyama K., Lipton S.A. (2006). Activation of the Keap1/Nrf2 pathway for neuroprotection by electrophilic phase II inducers. Proc. Natl. Acad. Sci. USA.

[113] Zou J., Wang R., Yu J., Chen X., Zhao Q., Li Y., Xu Y. (2025). Carnosic acid alleviated periodontitis by inhibiting ferroptosis via the Nrf2/GPX4 pathway. BMC Oral Health.

[114] Xu Y., Chu Y., Yang W., Chu K., Li S., Guo L. (2024). BML-111 inhibit H2O2-induced pyroptosis and osteogenic dysfunction of human periodontal ligament fibroblasts by activating the Nrf2/HO-1 pathway. BMC Oral Health.

[115] Wu S., Jia W., He H., Yin J., Xu H., He C., Zhang Q., Peng Y., Chen X. (2023). A New Dietary Fiber Can Enhance Satiety and Reduce Postprandial Blood Glucose in Healthy Adults: A Randomized Cross-Over Trial. Nutrients.

[116] Tanabe M., Takahashi T., Shimoyama K., Toyoshima Y., Ueno T. (2013). Effects of rehydration and food consumption on salivary flow, pH and buffering capacity in young adult volunteers during ergometer exercise. J. Int. Soc. Sports Nutr..

[117] Hans R., Thomas S., Garla B., Dagli R.J., Hans M.K. (2016). Effect of Various Sugary Beverages on Salivary pH, Flow Rate, and Oral Clearance Rate amongst Adults. Scientifica.

[118] Mohideen K., Chandrasekar K., Ramsridhar S., Rajkumar C., Ghosh S., Dhungel S. (2023). Assessment of Oxidative Stress by the Estimation of Lipid Peroxidation Marker Malondialdehyde (MDA) in Patients with Chronic Periodontitis: A Systematic Review and Meta-Analysis. Int. J. Dent..

[119] Maciejczyk M., Zalewska A., Ładny J.R. (2019). Salivary Antioxidant Barrier, Redox Status, and Oxidative Damage to Proteins and Lipids in Healthy Children, Adults, and the Elderly. Oxid. Med. Cell. Longev..

[120] Gruden Š., Poklar Ulrih N. (2021). Diverse Mechanisms of Antimicrobial Activities of Lactoferrins, Lactoferricins, and Other Lactoferrin-Derived Peptides. Int. J. Mol. Sci..

[121] Roi A., Roi C.I., Negruțiu M.L., Riviș M., Sinescu C., Rusu L.-C. (2020). The Challenges of OSCC Diagnosis: Salivary Cytokines as Potential Biomarkers. J. Clin. Med..

[122] Jaedicke K.M., Preshaw P.M., Taylor J.J. (2016). Salivary cytokines as biomarkers of periodontal diseases. Periodontol. 2000.

[123] Pan W., Wang Q., Chen Q. (2019). The cytokine network involved in the host immune response to periodontitis. Int. J. Oral Sci..

[124] Wang Y., Andrukhov O., Rausch-Fan X. (2017). Oxidative Stress and Antioxidant System in Periodontitis. Front. Physiol..

[125] Harris R., Gamboa A., Dailey Y., Ashcroft A. (2012). One-to-one dietary interventions undertaken in a dental setting to change dietary behavior. Cochrane Database Syst. Rev..

[126] Woelber J.P., Reichenbächer K., Groß T., Vach K., Ratka-Krüger P., Bartha V. (2023). Dietary and Nutraceutical Interventions as an Adjunct to Non-Surgical Periodontal Therapy—A Systematic Review. Nutrients.

[127] (2025). Probiotics and oral health—Current understanding and latest evidence. Br. Dent. J..

[128] Archambault L.S., Dongari-Bagtzoglou A. (2022). Probiotics for Oral Candidiasis: Critical Appraisal of the Evidence and a Path Forward. Front. Oral Health.

[129] Schlagenhauf U., Rehder J., Gelbrich G., Jockel-Schneider Y. (2020). Consumption of *Lactobacillus reuteri*-containing lozenges improves periodontal health in navy sailors at sea: A randomized controlled trial. J. Periodontol..

[130] Schlagenhauf U., Jockel-Schneider Y. (2021). Probiotics in the Management of Gingivitis and Periodontitis. A Review. Front. Dent. Med..

[131] Sachelarie L., Scrobota I., Romanul I., Iurcov R., Potra Cicalau G.I., Todor L. (2025). Probiotic Therapy as an Adjuvant in the Treatment of Periodontal Disease: An Innovative Approach. Medicina.

[132] Bartha V., Exner L., Schweikert D., Woelber J.P., Vach K., Meyer A.-L., Basrai M., Bischoff S.C. (2022). Effect of the Mediterranean diet on gingivitis: A randomized controlled trial. J. Clin. Periodontol..

[133] Paczkowska-Walendowska M., Grzegorzewski J., Kwiatek J., Leśna M., Cielecka-Piontek J. (2025). Green Tea: A Novel Perspective on the Traditional Plant’s Potential in Managing Periodontal Diseases. Pharmaceuticals.

[134] Cheever V.J., Mohajeri A., Patel K., Burris R.C., Hung M. (2025). Impact of Free Sugar Consumption on Dental Caries: A Cross-Sectional Analysis of Children in the United States. Dent. J..

[135] Humphrey L.T., De Groote I., Morales J., Barton N., Collcutt S., Bronk Ramsey C., Bouzouggar A. (2014). Earliest evidence for caries and exploitation of starchy plant foods in Pleistocene hunter-gatherers from Morocco. Proc. Natl. Acad. Sci. USA.

